# Self-citation is the hallmark of productive authors, of any gender

**DOI:** 10.1371/journal.pone.0195773

**Published:** 2018-09-26

**Authors:** Shubhanshu Mishra, Brent D. Fegley, Jana Diesner, Vetle I. Torvik

**Affiliations:** 1 School of Information Sciences, University of Illinois at Urbana-Champaign, Champaign, IL 61820, United States of America; 2 Illinois Informatics Institute, University of Illinois at Urbana-Champaign, Urbana, IL 61801, United States of America; Leiden University, NETHERLANDS

## Abstract

It was recently reported that men self-cite >50% more often than women across a wide variety of disciplines in the bibliographic database JSTOR. Here, we replicate this finding in a sample of 1.6 million papers from Author-ity, a version of PubMed with computationally disambiguated author names. More importantly, we show that the gender effect largely disappears when accounting for prior publication count in a multidimensional statistical model. Gender has the weakest effect on the probability of self-citation among an extensive set of features tested, including byline position, affiliation, ethnicity, collaboration size, time lag, subject-matter novelty, reference/citation counts, publication type, language, and venue. We find that self-citation is the hallmark of productive authors, of any gender, who cite their novel journal publications early and in similar venues, and more often cross citation-barriers such as language and indexing. As a result, papers by authors with short, disrupted, or diverse careers miss out on the initial boost in visibility gained from self-citations. Our data further suggest that this disproportionately affects women because of attrition and not because of disciplinary under-specialization.

## Introduction

Citing one’s own work is common practice, an essential part of scientific communication [1] that reflects the accumulative nature of research [2, 3], but it can be viewed negatively and is commonly discounted or penalized in impact metrics [4, 5]. The bibliometrics literature is rich in barriers and motivations for citation preferences [6–8], many of which overlap for self-citations [9] and vary e.g., by collaboration size [10]. Yet, what encourages authors to self-cite is not well understood. A recent study in particular [11–13] has gained high profile attention [14–17] in reporting that men self-cite >50% more often than women, an effect that is consistent across a wide variety of disciplines and has grown over time, reaching its peak of 70% in recent years. These effects are indeed jaw-dropping, align with gender stereotypes [18, 19], and imply that women may be severely disadvantaged because self-citations are not just additive but also attract other citations [20]. King et. al [13] offer a list of five possible explanations and “…consider it likely that several may contribute to the gender gap…” but are unable to test any of them for JSTOR. However, they confirm that, number 4 on their list, “Men publish more papers, particularly earlier in their career, and therefore have more to cite.” explains the gender-gap in its entirity for the Social Science Research Network (SSRN) pre-print repository, with the caveat that SSRN authors “…are not representative of academics, more generally.”.

It is well known that gender is often subject to confounding as in the pay-gap [21, 22] and productivity-gap [23], and that gender distributions in science vary dramatically across subject matter [24], geography [25], career length, position and productivity [26], and time [27, 28]. The effect of gender on self-citation could become negligible or even reverse after accounting for confounding factors. The reversal of an effect would be evidence of Simpson’s paradox [29, 30], of which the study of gender bias in UC Berkeley’s graduate admissions serves as a classic example [31]. A case study of archeology [32] found that author age has a strong effect on self-citation while gender is weak, in a carefully crafted sample of 285 articles analyzed using linear regression. Furthermore, the methods for citation parsing [33], identifying self-citations, and imputing gender are nontrivial and introduce errors, if not bias, into the overall analysis. For example, King et al.’s [13] analysis is US-centric (e.g., all English and Italian *Andrea’s* are labeled female while all *Shubhanshu’s* and *Vetle’s* are excluded), and Larivière et al. [25] reported highly assymetric error rates (≈ 13% errors for their female labels versus ≈ 1% for male labels) in an effort to cover names worldwide.

Here, we try to replicate this gender effect in PubMed, covering biomedicine broadly, while accounting for potential confounds and assessing the robustness of recently developed techniques for worldwide gender imputation [27, 34, 35]. We present a probabilistic model of self-citation based on over 1.6 million papers with 2 or more authors in PubMed during 2002-2005, with nearly 41.6 million citations of which 5.5 million (13.2%) are self-citations by at least one of the authors. The model captures the extent to which gender influences self-citations while controlling for other features of the papers and their disambiguated authors [36, 37].

## Materials and methods

### Two citation datasets with imputed gender: First and last authors

The contributions of each author on a multi-author paper systematically vary with their byline positions. Typically the first-listed author has less experience and does the most work, while the last-listed author has more experience and acts in a supervisory role, particularly in biomedicine. In order to account for this variability, we created two separate datasets: one for all first-listed authorships and one for all last-listed authorships. Each dataset is based on the 1.6 million PubMed papers with 2 or more authors published during 2002-2005 and with one or more references extracted from PubMed Central, Thomson Reuters’ Web of Science MEDLINE version, or Microsoft Academic Graph. The temporal restriction greatly improves gender imputation and author name disambiguaton because the National Library of Medline (NLM) started recording authors’ first names in MEDLINE in 2002. Overall, each dataset contains 41.6 million citation instances, of which 2.0 million (4.9%) are self-citations by the first author and 3.6 million (8.6%) are self-citations by the last author. There are 824 thousand unique first authors (with an average of 1.9 papers/author) and 539 thousand unique last authors (with an average of 3.0 papers/author) according to the Author-ity 2009 dataset, which has an overall disambiguation accuracy of 98% [36–38]. Disambiguation helps distinguish self-citations from other citations (even when an author has published under variant names, and when different authors with the same name cite each other), characterize each author’s publication history, and it improves the coverage of gender imputations because their first name need only be present on one of their papers. This paper focuses on first and last authors but includes a partial analysis of a third, slightly smaller dataset for middle authors, represented by all second-listed authors on the subset of papers with three or more authors, to confirm that the overall results do not differ significantly.

Gender is imputed as Male, Female, or Unknown using Genni 2.0 + Ethnea [27, 34, 35] which covers names worldwide and is ethnicity-sensitive. That is, it can make accurate predictions even for names that are rare in the USA but common elsewhere (such as *Shubhanshu* and *Vetle*) and it makes use of Ethnea’s ethnicity prediction to resolve genders for some names that are unisex worldwide but gender-specific regionally (e.g., English *Andrea’s* are labeled female and Italian *Andrea’s* are labeled male). Table 1 shows the improved coverage and potential bias-reduction compared to US Social Security Administration (SSA) data based gender assignment across a variety of ethnicities. Only the top 13 most common ethnicities (and UNKNOWN) are included while the remaining ethnicities were pooled and labeled OTHER. Overall, Genni and SSA predictions rarely disagree except for French and Italian names, where they disagree on more than 3% of authorships. As expected, Genni has higher coverage (e.g., 88.6% vs. 74.6% of all last authorships), more so for non-English names such as Nordic (96.3% vs. 65.7%) or Indian (68.3% vs. 45.0%) and less so for English names (95.4% vs. 91.7%). The lack of overall coverage is largely due to a large number of Chinese names that are difficult to classify (67.2% of 150k Chinese first authorships are labeled Unknown). Genni provides a slightly lower estimate of the overall Female proportion (33.0% of first authorships and 19.2% of last authorships) compared to SSA’s (34.8% of first authorships and 20.7% of last authorships). However, these proportions vary dramatically across byline position and ethnicities (ranging from 6.1% Japanese Female last authorships to 44.5% Slav Female first authorships). Taken together, this suggests that ethnicity and byline position are important covariates of gender in bibliometric studies broadly. In the spirit of data sharing and encouraging reproducibility, the Author-ity Exporter [39] web-interface was created to permit any user to search, browse, and export data from the annotated authors and papers in the Author-ity dataset. Furthermore, the subset of data based on PubMed Central is also shared [40].

**Table 1:** Comparison of gender proportions by using SSA data (with a 95% cut-off) versus Genni 2.0, aggregated by ethnicity. U denotes the percentage of authorships labelled Unknown, %F denotes the percentage of female authorships among male and female authorships, and G = SSA denotes the percentage of male and female SSA predictions that match the Genni predictions.

Ethnicity	First Author							Last Author						
	Proportion	Total	Genni		SSA		G = SSA	Proportion	Total	Genni		SSA		G = SSA
			U	%F	U	%F				U	%F	U	%F	
ENGLISH	25.4	411,560	5.7	33.5	9.5	33.2	100.0	32.9	532,957	4.6	20.2	8.3	19.9	100.0
GERMAN	10.1	163,416	3.7	26.9	16.7	28.2	100.0	11.0	178,641	3.0	13.1	16.7	13.8	100.0
HISPANIC	7.7	124,765	7.2	43.4	16.2	42.6	99.6	6.7	109,191	6.8	28.6	16.5	28.0	99.4
CHINESE	9.3	150,709	67.2	29.2	82.4	33.2	99.5	6.0	97,880	63.6	22.5	81.2	24.5	98.8
JAPANESE	8.7	140,021	12.2	16.8	39.2	21.3	99.6	8.5	138,282	13.4	6.1	42.6	7.8	99.7
SLAV	4.1	66,162	7.8	44.5	26.9	48.0	99.2	3.1	49,543	8.8	30.6	30.4	33.2	98.8
FRENCH	6.7	108,456	5.3	37.1	17.5	42.3	96.1	7.0	113,352	4.0	20.6	18.5	30.9	90.5
ITALIAN	5.4	87,438	3.0	37.7	10.2	41.1	96.9	5.1	82,474	3.0	21.5	10.7	24.2	97.4
INDIAN	4.3	69,373	29.6	31.0	50.7	35.9	99.6	3.1	49,801	31.7	21.5	55.0	26.0	99.6
NORDIC	3.8	62,213	4.9	44.0	30.4	48.0	98.0	4.2	68,099	3.7	22.4	34.3	26.2	98.3
ARAB	3.0	48,540	17.2	22.5	34.8	22.9	99.6	1.9	30,980	17.1	16.7	36.0	17.0	99.6
DUTCH	2.9	46,419	9.5	35.3	33.5	38.6	99.3	3.2	52,106	7.6	13.6	36.1	15.8	99.6
KOREAN	2.2	36,143	52.6	36.3	85.7	36.8	98.3	1.7	27,018	59.7	22.5	84.3	23.7	99.1
UNKNOWN	0.2	3,440	19.4	43.2	25.7	44.6	99.9	0.2	2,976	15.4	28.7	23.0	29.2	100.0
OTHER	6.1	99,344	16.0	31.2	40.2	29.9	99.3	5.2	84,699	13.3	21.0	37.5	19.7	99.3
OVERALL	100.0	1,617,999	15.0	33.0	29.0	34.8	99.2	100.0	1,617,999	11.4	19.2	25.4	20.7	98.9

### Explanatory features modeled

Each observation (instance) in the datasets captures features about a particular citation and the paper in which it appears. More concretely, features include aspects of (a) a given paper, (b) the paper it cites, (c) similarity or nearness between the paper and the cited paper, and (d) the first or last author, namely professional age, gender, ethnicity, and country of affiliation (inferred using MapAffil [41, 42]). The features used for modeling are listed in Table 2. The distribution of instances in the two datasets for a select group of categorical features are shown in Table 3.

**Table 2:** Descriptions of all the explanatory features.

Feature	Description
*gender*	is the gender of the author in question as predicted by Genni 2.0 [27, 34, 35]. Each author is labeled as one of the following: Female, Male, or Unknown.
*age*(*pub count*)	is the age of the author as measured by the number of papers published in the years prior to the article in question.
*ethnicity*	is the ethnicity of the author as predicted by Ethnea [34]. Each author can have one (or a mixture of two, equally weighted) of the following ethnicities: ARAB, CHINESE, DUTCH, ENGLISH, FRENCH, GERMAN, HISPANIC, INDIAN, ITALIAN, JAPANESE, KOREAN, NORDIC, OTHER, SLAV, and UNKNOWN.
*country*	is the country of affiliation of the first-listed author on the article in question, as inferred by MapAffil [41, 42]. Each article is assigned one of the following countries: Australia, Canada, China, France, Germany, India, Italy, Japan, Netherlands, Other, Spain, Sweden, UK, Unknown, or USA.
*collabortion size*	is the number of authors on the article in question, capped at 20.
*language*	is 1 if the article was written in English as tagged in MEDLINE [43], 0 otherwise.
*reference count*	is the total number of references listed on the article in question.
*MeSH count*	is the number of MeSH terms (and all their unique ancestors in the MeSH tree structure) as assigned in MEDLINE.
*novelty score*	is the number of prior papers for the youngest MeSH term assigned to the article in question (the so-called “volume novelty” in [44, 45]).
*pub type*	is the publication type(s) of the referenced article as tagged in MEDLINE [43]. Each article can have one or more of following: “journal article”, “case report”, “review article”, and “letter/editorial/comment”.
*venue*	is encoded by indicating whether the article and its reference were published in the same or similar journal as captured by the exact name match and the implicit journal score [46] which is similar to author odds ratio [47].
*timelag*	is the difference in publication years between the articles in question and its reference
*ref. language*	is 1 if the referenced article was written in English as tagged in MEDLINE [43], 0 otherwise.
*ref. citation count*	is the number of citations received by the referenced article prior to the citation in question.
*ref. MeSH count*	is the number of MeSH terms (and all their unique ancestors in the MeSH tree structure) assigned to the referenced article.
*ref. novelty score*	is the number of prior papers for the youngest MeSH term assigned to the referenced article (the so-called “volume novelty” in [44, 45]).
*ref. pub type*	is the publication type of the referenced article as tagged in MEDLINE [43]. Each article can have one or more of following: “journal article”, “case report”, “review article”, and “letter/editorial/comment”.

**Table 3:** Distribution (in percentage) of 41.6 million references (from 1.6 million articles with 2 or more authors published during 2002-2005) across select categorical features.

Features	First Author	Last Author
*language* = *English*	98.2	98.2
*pub type* = *Journal Article*	98.6	98.6
*pub type* = *Review*	21.7	21.7
*pub type* = *Case Report*	3.5	3.5
*pub type* = *Letter*/*Editorial*/*Comment*	1.4	1.4
*ref. language* = *English*	99.5	99.5
*ref. pub type* = *Journal Article*	98.2	98.2
*ref. pub type* = *Review*	14.2	14.2
*ref. pub type* = *Case Report*	3.3	3.3
*ref. pub type* = *Letter*/*Editorial*/*Comment*	1.8	1.8
*same journal*	7.8	7.8
*is self*–*citation*	4.9	8.6

Several of these features capture known motivations for self-citation narrowly, and citation broadly: (a) prior citations: authors tend to cite papers that have previously received citations; (b) time: one cannot (typically) cite papers that do not yet exist and self-citations might appear sooner [48]; (c) publication count: an author cannot self-cite if they don’t have any published (or working) papers; (d) language [43]: one is less likely to cite papers that one cannot understand [49]; (e) disciplinary barriers and topical diversity (encoded by indicating whether the article and its reference were published in the same or similar journal as captured by the exact name match and the implicit journal score [46] which is similar to author odds ratio [47]): academic careers often depend on intra- vs. inter-disciplinary citations and scientists who jump from one topic to another are less likely to cite themselves; (e) accessibility and discoverability: what one cites may be limited by how easy it is to find and obtain physically [50]; (f) publication type [43]: one may cite *writing*, not necessarily *research*; literature reviews tend to cited more often; (g) novelty or topical narrowness: articles on young topics tend to be cited more often [44]; (h) collaboration size: as the number of co-authors increases, the individual opportunity for self-citation may decrease.

## Results

### Simple characterizations of age-normalized self-citation rates

Before presenting the full model, we start with simple characterizations of self-citation as a function of author age and gender using focused subsets of the datasets. First, the data are restricted to papers with 10 to 60 references, which will focus the characterization on primary research articles and exclude papers with unusual citation patterns such as short comments and letters, as well as long review papers. Second, the data are restricted to instances where the gender is known (labeled Male or Female). This leaves 26.2 million first author references, and 27.5 million last author references.

**Fig 1:**
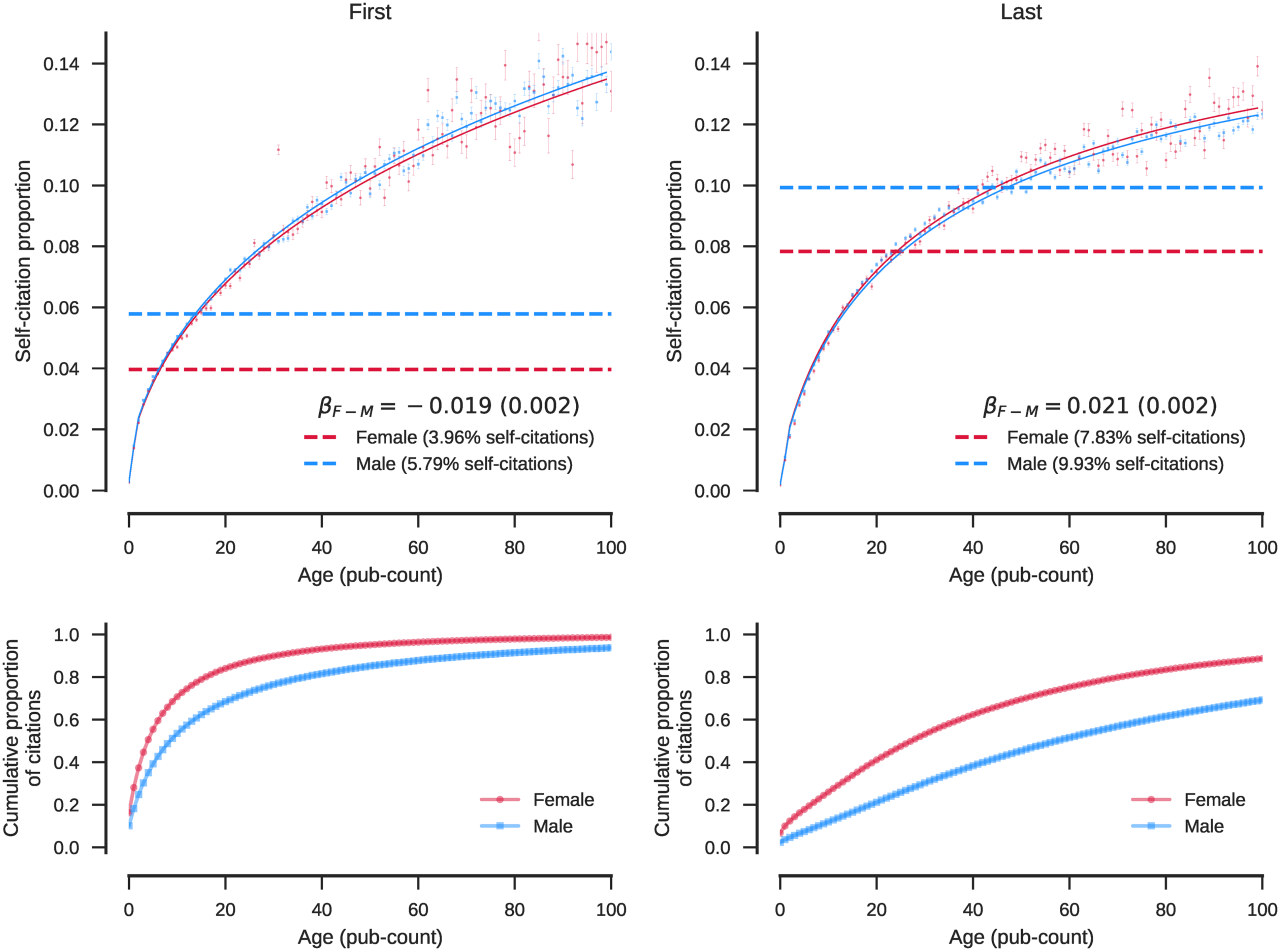
Self-citation rates as functions of author age as measured by prior publication count (top panels). The horizontal lines show the overall self-citation rates. The bottom panels show the cumulate distributions of author age.

The bottom panels in Fig 1 show that women tend to be younger (as measured by prior publication count) both as first-listed and last-listed authors, and thus have fewer opportunities for self-citation. The top panels in Fig 1 show the overall relationships between self-citation rate and author age where the self-citation rate is modeled as a logistic regression function of gender and author age (pub count). The horizontal lines show that overall self-citation rates differ dramatically between women and men. Men self-cite 46% more often than women as first authors (5.79% vs. 3.95%), and 27% more often as last authors (9.93% vs. 7.83%). However, the logistic regression curves that account for authors’ prior publication counts are nearly identical. It appears that the age-normalized self-citation rate among men is about 1.9% (SE = 0.2%) higher as first authors, but 2.1% (SE = 0.2%) lower as last authors. However, these differences are within the error rates of the techniques used to disambiguate author names and predict gender, so we cannot say for sure that these differences are real. This preliminary characterization reveals that there are more important factors than gender that govern self-citation.

S1 Fig shows that the gender effect for middle authors, as represented by the second author on papers with three or more authors, is similar to that of last authors. The age-normalized self-citation rate among men is about 2.2% (SE = 0.2%) lower than among women. Middle authors self-cite less overall (4.67% for men and 3.32% for women) compared to first and last authors. However, this effect can be explained, at least partly, by the fact that the first and last author datasets include two-author papers, where they tend to self-cite more. Excluding the two author papers reduced the dataset from 1.6 to 1.3 million papers and 41.6 to 34.0 million references.

For a secondary part of this simple characterization, the top-most prolific journals were selected (each with at least 20,000 references). These journals capture variations within and between three broad subject areas: general science, biology, medicine as shown in Table 4. In general, the journal-subsetting yields bigger effects but much less statistical significance. It should also be noted that the sample sizes appear large because each reference is counted separately. The total number of articles is much smaller than the total number of references (a typical paper has about 30 references), and the number of unique female (or male) authors of a given age is a fraction of the number articles. In other words, the degrees of freedom are inflated and the reported p-values are likely underestimated. The high variability between observed proportions versus model expectations is illustrated in the S2 Fig (science journals), S3 Fig (biology journals), and S4 Fig (medicine journals). After adjusting for age, men tend to self-cite more as first authors but less as last authors, in the majority of the journals, a finding that is consistent with Fig 1. The majority individual journal effects are statistically insignificant (with p-value > 0.05), particularly among medical journals. After adjusting for age, there is one journal where men self-cite more as both first and last authors (Biochem J), and one journal (Cancer Res) where women self-cite more as both first and last authors. These effects may be the result of additional confounding factors not considered in this preliminary analysis but are presented next.

**Table 4:** Gender effects for selected journals using a simple model with author age (pub count) only.

Category	Journal	First Author				Last Author			
		references	*β*_*F*−*M*_	SE	p-val	references	*β*_*F*−*M*_	SE	p-val
Science	PNAS	298,356	-0.067	0.020	0.001	334,968	-0.016	0.015	0.297
	Ann N Y Acad Sci	52,540	-0.035	0.033	0.288	54,224	0.214	0.034	0.000
	Nature	46,138	-0.162	0.054	0.003	50,625	0.028	0.044	0.531
	Science	41,328	-0.154	0.053	0.004	45,043	0.066	0.041	0.107
Biology	J Biol Chem	676,859	-0.095	0.013	0.000	758,553	0.009	0.010	0.348
	Biochemistry	182,433	-0.036	0.024	0.143	204,527	0.030	0.017	0.084
	J Virol	155,081	-0.063	0.025	0.013	172,265	0.024	0.018	0.185
	Biochim Biophys Acta	104,434	-0.028	0.029	0.344	110,138	0.003	0.025	0.915
	J Bacteriol	102,012	-0.020	0.032	0.535	109,737	0.082	0.022	0.000
	Nucleic Acids Res	98,322	-0.107	0.035	0.002	104,933	-0.051	0.027	0.061
	FEBS Lett	94,021	-0.086	0.031	0.005	99,364	0.007	0.027	0.805
	Biochem J	91,576	-0.176	0.034	0.000	97,174	-0.075	0.026	0.004
	Mol Cell	33,538	-0.090	0.067	0.182	39,524	-0.203	0.045	0.000
	Cell	32,399	-0.200	0.076	0.008	37,042	-0.089	0.050	0.073
	Adv Exp Med Biol	21,625	-0.081	0.053	0.124	22,072	0.101	0.056	0.070
	Bioinformatics	20,756	-0.080	0.103	0.437	23,014	0.009	0.082	0.912
Medicine	J Immunol	208,354	-0.021	0.024	0.389	228,129	-0.017	0.017	0.324
	Blood	140,887	-0.041	0.028	0.140	152,394	0.000	0.022	0.984
	Cancer Res	131,329	0.056	0.029	0.057	149,313	0.051	0.022	0.018
	Brain Res	100,389	-0.071	0.031	0.025	108,379	0.004	0.028	0.882
	Circulation	92,220	-0.020	0.036	0.575	98,741	-0.051	0.035	0.143
	Clin Cancer Res	91,687	0.057	0.035	0.101	96,551	0.050	0.031	0.113
	J Clin Oncol	61,722	0.069	0.041	0.093	62,049	0.056	0.041	0.176
	J Am Coll Cardiol	50,523	-0.070	0.061	0.248	52,579	0.124	0.057	0.030
	J Urol	49,314	0.274	0.063	0.000	50,379	0.099	0.063	0.113
	JAMA	33,674	0.164	0.050	0.001	34,651	-0.012	0.055	0.825
	Gut	31,027	0.000	0.065	1.000	32,663	0.028	0.069	0.683
	N Engl J Med	30,970	0.068	0.061	0.270	31,971	0.030	0.065	0.637
	Lancet	26,344	-0.074	0.059	0.209	26,301	-0.061	0.065	0.348
	BMJ	23,987	0.170	0.065	0.009	24,438	0.123	0.073	0.091

### A probabilistic model of self-citation

The observed citations were modeled using logistic regression

$$\begin{array}{c} Pr\left(is\_self\_citation=1\right)=\frac{1}{1+e^{-\beta_{0}-\beta_{1}x_{1}-\beta_{2}x_{2}...-\beta_{n}x_{n}}} \end{array}$$

where

$$\begin{array}{c} x_{1},x_{2},...,x_{n} \end{array}$$

denote the explanatory features described in Table 2 and the binary outcome indicates whether or not an observation is a self-citation. The use of logistic regression as the modeling framework is justified by the fits shown in S5 Fig, particularly the linearity assumption. Local intercepts, indicators, and polynomials were introduced to capture non-linearities and facilitate a closer fit to the data. The complete models of self-citation for first authors and last authors are detailed in Table 5. Although the model is inadequate for predicting whether any given citation was a self-citation or not, given its low precision and recall (S6 Fig), prediction was not the aim of this study. Rather, the purpose is to measure the degree to which each of the encoded features influence self-citations, as captured by the effects (or weights) listed in this table. Note that one of columns includes simple models of individual categories for first authors, to be compared to the full first author model so that the degree of confounding can be assessed.

**Table 5:** Models of self-citation behavior of first and last authors based on 41.6 million references from 1.6 million articles with 2 or more authors published during 2002-2005.

Predictor^†^	First author effects, simple models^‡^	First author effects, complete model	Last author effects, complete model
Intercept	—	-1.957 (0.031)	-2.790 (0.027)
**gender (vs. Unknown)**			
Female	0.021 (0.003)	-0.029 (0.003)	0.026 (0.003)
Male	0.451 (0.002)	-0.039 (0.003)	-0.002 (0.003) X
**age**			
*log*_10_(*pub count* + 1)	1.440 (0.007)	1.610 (0.007)	2.172 (0.008)
*log*_10_(*pub count* + 1)^2^	-0.119 (0.002)	-0.091 (0.003)	-0.340 (0.002)
*pub count* = 0	-1.375 (0.009)	-1.421 (0.009)	-1.232 (0.016)
*pub count* = 1	-0.210 (0.005)	-0.236 (0.005)	-0.187 (0.011)
**ethnicity (vs. ENGLISH)**			
ARAB	-0.470 (0.006)	-0.055 (0.006)	-0.085 (0.006)
CHINESE	-0.444 (0.003)	0.046 (0.004)	0.034 (0.004)
DUTCH	-0.068 (0.004)	0.030 (0.007)	-0.004 (0.005) X
FRENCH	-0.205 (0.003)	-0.074 (0.004)	-0.038 (0.003)
GERMAN	-0.056 (0.003)	-0.014 (0.004)	0.062 (0.003)
HISPANIC	-0.261 (0.003)	0.053 (0.004)	0.082 (0.003)
INDIAN	-0.418 (0.004)	-0.064 (0.005)	0.020 (0.005)
ITALIAN	0.084 (0.003)	-0.060 (0.006)	0.026 (0.004)
JAPANESE	0.018 (0.003)	-0.102 (0.006)	-0.048 (0.006)
KOREAN	-0.656 (0.007)	-0.187 (0.007)	-0.076 (0.006)
NORDIC	0.037 (0.004)	0.136 (0.005)	0.070 (0.004)
OTHER	-0.362 (0.004)	-0.181 (0.004)	-0.057 (0.003)
SLAV	-0.103 (0.004)	0.044 (0.005)	0.032 (0.004)
UNKNOWN	-0.318 (0.017)	0.013 (0.018) X	-0.046 (0.014)
**country (vs. USA)**			
Australia	0.004 (0.005)	0.088 (0.005)	-0.099 (0.004)
Canada	-0.121 (0.004)	-0.004 (0.004) X	-0.069 (0.003)
China	-0.751 (0.009)	-0.412 (0.010)	-0.432 (0.007)
France	-0.133 (0.004)	-0.044 (0.005)	-0.116 (0.004)
Germany	0.029 (0.003)	0.024 (0.004)	-0.169 (0.003)
India	-0.359 (0.009)	-0.044 (0.011)	-0.206 (0.008)
Italy	0.268 (0.003)	-0.101 (0.007)	-0.298 (0.005)
Japan	0.154 (0.003)	0.267 (0.006)	-0.093 (0.006)
Netherlands	-0.022 (0.005)	0.068 (0.007)	-0.179 (0.005)
Other	-0.124 (0.002)	-0.064 (0.003)	-0.213 (0.002)
Spain	-0.002 (0.005)	-0.122 (0.006)	-0.184 (0.005)
Sweden	0.113 (0.005)	0.107 (0.007)	-0.083 (0.005)
UK	-0.050 (0.003)	-0.005 (0.003) X	-0.090 (0.002)
Unknown	0.091 (0.006)	-0.045 (0.006)	-0.159 (0.005)
**author count**			
*author count* > 20	0.018 (0.014)	0.000 (0.015) X	-0.050 (0.013)
*log*_10_(*author count*)	-1.347 (0.016)	0.082 (0.018)	-0.444 (0.014)
*log*_10_(*author count*)^2^	0.941 (0.012)	-0.256 (0.013)	0.067 (0.010)
**language**			
ref. English	-0.456 (0.009)	-0.185 (0.010)	-0.031 (0.009)
English	0.488 (0.007)	0.649 (0.008)	0.701 (0.007)
**references**			
*log*_10_(*count*)	-1.977 (0.028)	-1.647 (0.034)	-2.221 (0.031)
*log*_10_(*count*)^2^	0.742 (0.020)	0.820 (0.023)	1.463 (0.022)
*log*_10_(*count*)^3^	-0.125 (0.004)	-0.229 (0.005)	-0.387 (0.005)
count = 1	-0.390 (0.019)	-0.300 (0.022)	-0.405 (0.021)
**MeSH**			
*log*10_10_(*ref. count* + 1)	0.832 (0.004)	-0.168 (0.006)	-0.092 (0.004)
*ref. count* = 0	1.978 (0.009)	-0.093 (0.015)	0.005 (0.011) X
*log*10_10_(*count* + 1)	-0.693 (0.004)	-0.170 (0.006)	-0.251 (0.004)
*count* = 0	-0.812 (0.009)	-0.667 (0.013)	-0.892 (0.010)
**novelty**			
*log*10_10_(*ref. score* + 1)	-0.329 (0.003)	0.095 (0.008)	0.048 (0.005)
*log*10_10_(*ref. score* + 1)^2^	0.079 (0.001)	-0.028 (0.001)	-0.022 (0.001)
*log*10_10_(*score* + 1)	-0.062 (0.001)	-0.106 (0.001)	-0.139 (0.001)
**pub type**			
ref = Case Report	-0.423 (0.006)	-0.637 (0.006)	-0.731 (0.005)
ref = Journal	0.103 (0.011)	0.328 (0.011)	0.324 (0.009)
ref = Letter	-0.044 (0.011)	-0.284 (0.011)	-0.292 (0.009)
ref = Review	-0.723 (0.003)	-0.783 (0.003)	-0.641 (0.002)
Case Report	-0.892 (0.006)	-0.940 (0.007)	-0.948 (0.005)
Journal	-0.219 (0.011)	0.266 (0.011)	0.229 (0.010)
Letter	0.468 (0.011)	-0.430 (0.011)	-0.343 (0.010)
Review	0.080 (0.002)	-0.079 (0.003)	-0.249 (0.002)
**venue**			
same journal	0.492 (0.003)	0.457 (0.003)	0.534 (0.002)
*log*_10_(*journal similarity* + 1)	0.214 (0.009)	0.063 (0.010)	0.126 (0.008)
*log*_10_(*journal similarity* + 1)^2^	0.050 (0.003)	0.006 (0.003) *	-0.050 (0.003)
*journal similarity* = 0	-0.178 (0.008)	-0.281 (0.008)	-0.296 (0.006)
**time lag**			
*time lag* < 0	-1.884 (0.015)	-0.556 (0.016)	-0.383 (0.015)
*log*_10_(*time lag* + 1)	-2.099 (0.013)	0.539 (0.015)	1.232 (0.011)
*log*_10_(*time lag* + 1)^2^	-0.275 (0.009)	-1.281 (0.010)	-1.402 (0.007)
*time lag* = 0	0.016 (0.005)	0.538 (0.006)	0.735 (0.005)
**ref. citation**			
*log*_10_(*count* + 1)	-0.769 (0.006)	-0.443 (0.006)	-0.079 (0.004)
*log*_10_(*count* + 1)^2^	-0.199 (0.002)	-0.170 (0.003)	-0.169 (0.002)
count = 0	0.161 (0.004)	0.127 (0.004)	0.240 (0.003)

^†^Format: logit (SE) signif., where X = *p* ≥ 0.05, * = *p* < 0.05, . = *p* < 0.01, and *p* < 0.001 otherwise.

^‡^Each category represents a simple model (with only one predictor); intercepts not shown.

A few additional aspects of the data and its encoding as well as model specification and model interpretation deserve mention:

- Because language, ethnicity, and affiliation are tightly correlated, given the multi-ethnic composition of modern societies, our approach to language is simplistic. For example, we test only whether the language of the citing or cited papers are English, including translations. Thus, the results of this study cannot be the basis for a claim that one ethnicity self-cites more than another, although the results may offer clues about culture categorically. Among other features, journal similarity captures author links between journals; the higher the score, the more likely self-citation, reflecting a broad rather than narrow pattern of citation.
- The novelty of an article or reference is determined by the relative frequency of its associated MeSH terms [44, 45]. The novelty scores *score* and *ref. score* follow a similar encoding, where lower scores indicate more novel papers.
- Due to the indexing policies of the National Library of Medicine (NLM) about PubMed for the period considered, only the first author’s affiliation is retained and, for this study, assigned to the last author as well. Thus, for the model of last authors, affiliation (country) and ethnicity, which is dependent to a degree on affiliation, is less accurate and not as tightly coupled with ethnicity and language as for the model of first authors.

### Gender effect

**Fig 2:**
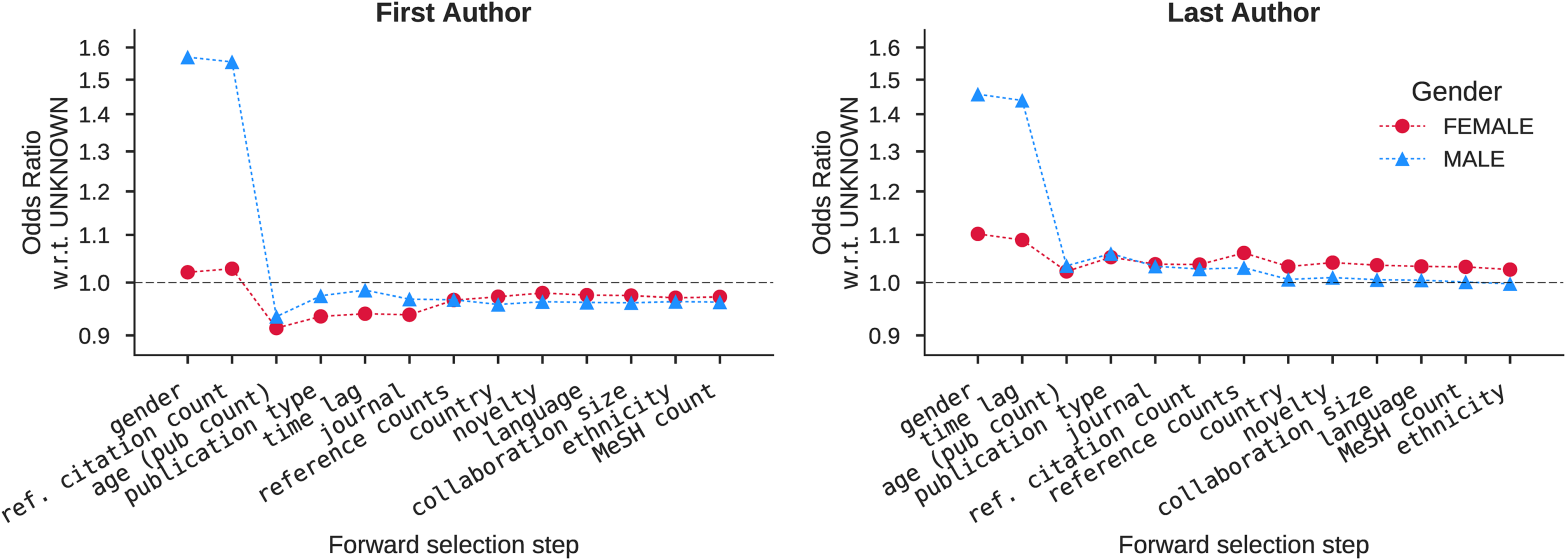
Change in effect of gender at each model-fitting step. The sub-figures show the contribution of gender at each step in the iterative process of fitting and evaluating combinations of factors; only the model at the final step is the best-fitting among them. In both models, confounding factors ultimately minimize the effect of gender in self-citation; the most influential of them is author’s publication count (note Table 6). Y-axis is on log scale.

A gender-effect does exist but its magnitude and relative importance depend on what other factors a model includes (see Fig 2). For first authors, when considering gender only, males self-cite ≈ 54% more often than females, a percentage congruent with the findings of [11, 51]. However, when controlling for other factors, the difference becomes negligible (Table 5; exp(0.451 − 0.021) = 1.54 vs. exp(−0.04 + 0.03) = 0.99). Sign flips between the individual model of gender and the complete model provides evidence of extreme confounding (Simpson’s paradox), which means that conclusions drawn from analysis of the individual model are unreliable [29, 30].

In the presence of confounding factors, the models show that gender has little affect on self-citation after-all. Gender is the least explanatory of all the features modeled for both first and last authors when factors are ranked by contribution (Table 6). For first authors, a plot of the self-citation odds-ratio of male and female gender with respect to the unknown gender shows that the known gender’s influence effectively disappears after inclusion of (a) prior citation count of the reference before being cited by the paper, (b) author’s publication count before source paper’s published year, and (c) publication type; for last authors, time lag (the number of years between the source and the cited paper). Factoring in author’s publication count results in the largest reduction of the gender effect (Fig 2). Moreover, the relationship between male and female self-citing behavior is effectively identical when affiliation is added for first authors (step 7) and when journals is added for last authors (step 4); the relationship reverses and diverges thereafter, suggesting that females self-cite more often than males when all else is equal. However, the effect is negligible and probably below the level detectable using our methods.

**Table 6:** Fit statistics for individual and accretive models of self-citation based on 41.6 million references from 1.6 million articles with 2 or more authors published during 2002-2005. The best-performing model at each step is the one with the largest log-likelihood (LL); only the highest-ranking of which are shown in steps 2 and following. Models comprise the predictors from the best-performing models in all previous steps along with the newly added category indicated by the plus sign (+). AUC (Area Under the receiver operating characteristic Curve), given as a percentage, roughly measures the accuracy of estimated probabilities. The number of terms in the model is denoted by nf, excluding intercept.

	First authors				Last authors			
Step	Feature	LL(10^5^)	nf	AUC	Feature	LL(10^5^)	nf	AUC
1	ref. citation count	-74.1	3	72.9	time lag	-117.4	4	65.1
	age (pub count)	-74.5	4	71.9	ref. citation count	-117.8	3	64.6
	time lag	-74.9	4	71.4	age (pub count)	-118.4	4	62.5
	venue	-78.8	4	61.7	venue	-119.9	4	60.0
	pub type	-80.0	8	55.4	pub type	-120.8	8	57.7
	reference count	-80.2	4	56.3	reference count	-121.8	4	53.7
	gender	-80.3	2	55.2	MeSH count	-122.0	4	54.8
	MeSH count	-80.3	4	55.1	country	-122.2	14	53.5
	ethnicity	-80.4	14	54.8	ethnicity	-122.2	14	53.4
	country	-80.6	14	52.9	gender	-122.2	2	53.0
	novelty	-80.6	3	53.5	novelty	-122.3	3	53.3
	author count	-80.7	3	51.6	language	-122.3	2	50.6
	language	-80.7	2	50.3	author count	-122.5	3	51.3
2	+ age (pub count)	-67.7	7	82.2	+ age (pub count)	-113.3	8	70.1
3	+ pub type	-66.5	15	83.2	+ pub type	-111.2	16	72.5
4	+ time lag	-65.4	19	84.2	+ venue	-109.6	20	74.0
5	+ venue	-64.6	23	84.8	+ ref. citation count	-108.3	23	75.2
6	+ reference count	-64.0	27	85.2	+ reference count	-107.9	27	75.6
7	+ country	-63.9	41	85.3	+ country	-107.7	41	75.7
8	+ novelty	-63.9	44	85.3	+ novelty	-107.6	44	75.9
9	+ language	-63.8	46	85.3	+ author count	-107.5	47	75.9
10	+ author count	-63.8	49	85.4	+ language	-107.4	49	76.0
11	+ ethnicity	-63.8	63	85.4	+ MeSH count	-107.3	53	76.0
12	+ MeSH count	-63.7	67	85.4	+ ethnicity	-107.3	67	76.1
13	+ gender	-63.7	69	85.4	+ gender	-107.3	69	76.1

### Factors affecting self-citation

**Fig 3:**
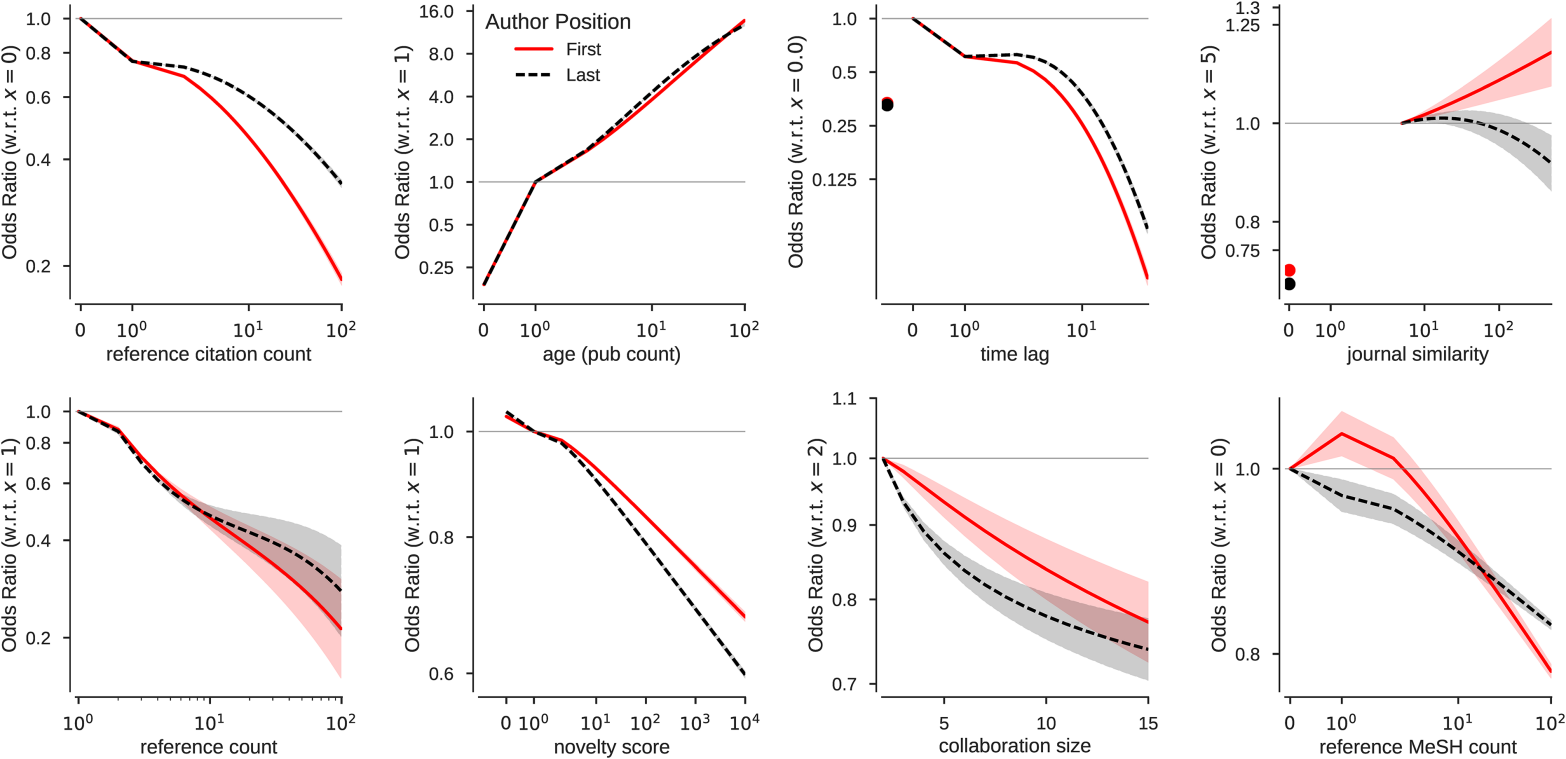
Change in odds with respect to mentioned values (in parentheses) of self-citation for select predictors of models of first and last authors. Shaded regions indicate 95% confidence intervals. Y-axis is on log scale.

**Fig 4:**
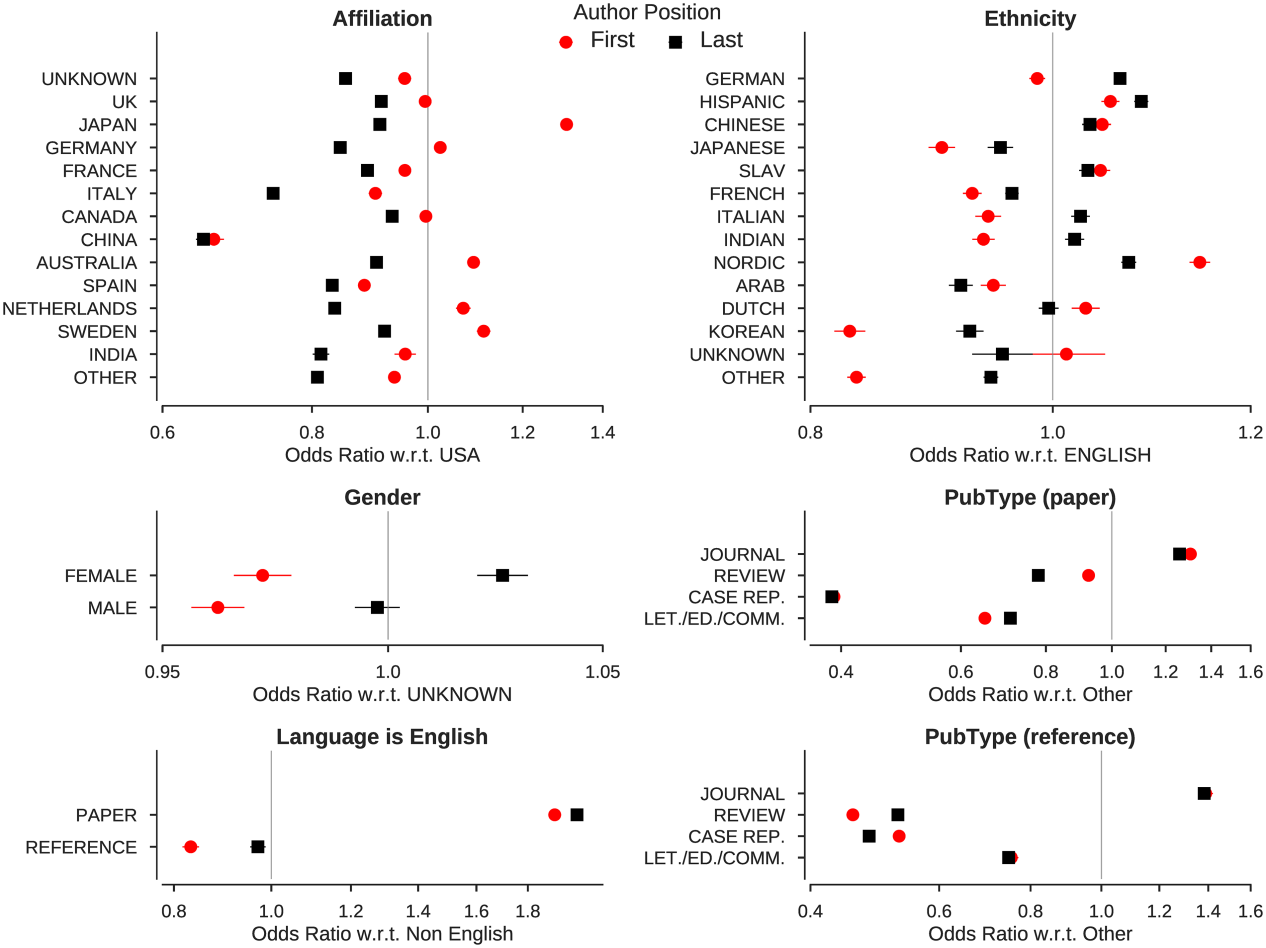
Change in odds with respect to mentioned values of self-citation for select predictors of models of first and last authors. Error bars indicate 95% confidence intervals. Among other interesting points, note that the likelihood of self-citation is least for last authors with non-USA affiliation, implying that self-citing is customary among USA authors. X-axis is on log scale.

The features that explain the most self-citation in both first author and last author models have more to do with opportunity, accessibility, and visibility than gender and culture (ethnicity, language, affiliation). The former aspects are illustrated in Fig 3, while the latter are illustrated in Fig 4, using related coefficients from the complete models detailed in Table 5.

Opportunity is a major factor driving self-citation. An author cannot legitimately self-cite without having produced work to cite; and a paper on a novel topic will have fewer papers to reference. The choice among citeable papers is bounded. Thus, the more self-authored papers one has available, the more opportunity one has for self-citation. The model captures this effect. Going from none to one prior paper increases the odds of self-citation 4-fold, while the increase is 5-fold going from 1 to 10 prior papers (Fig 3 and Table 5). Note that it is possible to self-cite with no prior papers, e.g., when the cited paper is published simultaneously or in the same year. Although rare, some cited papers are even published in future years e.g., due to extensive journal back-logs or review processes. When a paper has few references, each is more likely to be a self-citation compared to those with normal or a high number of references. Furthermore, the odds of self-citation are highest for first and last authors on 2-author papers; suggesting some limits in self-citing in the presence of more authors on the source paper.

Accessibility and visibility are surely driving forces of citations generally, and as such, might counteract self-citations. After all, our model is designed to distinguish self-citations from citations by others. The journal publication type has a 40% higher odds of self-citation than a review, while a reference to a journal article is 80% more likely to be a self-citation than a review article (see Fig 4). One is not likely to cite work that one cannot understand (due to language or discipline) or that is otherwise inaccessible (due to indexing issues or economic barriers such as pay-walls or firewalls). Culture and language both act as a barriers in this regard, limiting what can be cited by a particular audience. Language also reflects social norms, which are much more difficult to discern but tightly correlated with ethnicity and geography. A non English reference has 20% higher odds of being a self-citation, while an English paper has 90% higher odds of including a self-citation. The odds of self-citation for a first author in Japan are 30% higher than that in USA, while the odds for an first author in Italy are 10% less. Similarly, a first author with a Japanese name has 10% lower odds of self-citation compared to an author with an English name, while an author with an Italian name has 6% lower odds. Authors of with Nordic names self-cite more than any other ethnicity, regardless of gender—a behavior that would contravene Janteloven [52] if construed as self-promotion or an attempt to *“game the system”*; but perhaps language is a barrier for the Nordic too.

Citations increase the visibility of a paper and presumably the visibility of its author(s). This is self-evident; and so the result that prior citations of the reference is the most influential in the first author model is unsurprising. For first authors, a reference paper published in the same year as the paper citing it increases the odds of self-citation by 70%; for last authors, the same publication year doubles the odds. The effect of a multi-year difference is strongly tempered, meaning only recent references are more likely to be self-citations. What makes these particular results interesting is that, for both first and last authors, references with any prior citations decrease the odds of self-citation, more for first authors than last authors. Thus, for both first and last authors, the odds of self-citation increases when the reference is recent and has few if any citations. This corroborates the finding of Aksnes et. al [2], which states that most of the early citations of an article are self-citations; and might point towards the opportunistic nature of authors of bootstrapping the initial citations of their prior work through means of self-citations. The effect of gender is reduced when accounting for these additional factors, as evident from the dramatic change in odds of self-citation (Fig 2). This probabilistic model suggests that self-citation is more about opportunity, accessibility, and visibility than culture or gender. These results in the self-citation model vary only sightly when the analysis is conducted on a filtered data set where the country of affiliation is USA, ethnicity is ENGLISH, source language is ENGLISH, number of references is at least 10 (and at-max 100 for first authors and 50 for last authors), and source publication type is JOURNAL. The coefficient for female gender remains same, while the coefficient for male authors increases slightly for both first and last authors while becoming non-significant (p >0.05). This indicates that the results are robust under the consideration of the largest portion of the data set (See Table 7).

**Table 7:** Comparison of full model (based on all 41.6 million references from 1.6 million articles with 2 or more authors published during 2002-2005) with filtered models (26.2 million references for first authors, and 27.5 million for last authors).

	First Author^†^		Last Author^†^	
	Full	Filtered	Full	Filtered
Intercept	-1.96 (0.031)	-3.54 (0.482)	-2.79 (0.027)	-0.49 (0.695) X
**ref. citation**				
*count* = 0	0.13 (0.004)	0.16 (0.011)	0.24 (0.003)	0.29 (0.009)
*log*_10_(*count* + 1)	-0.44 (0.006)	-0.14 (0.016)	-0.08 (0.004)	0.22 (0.012)
*log*_10_(*count* + 1)^2^	-0.17 (0.002)	-0.18 (0.006)	-0.17 (0.002)	-0.20 (0.004)
**age**				
*pub count* = 0	-1.42 (0.009)	-1.24 (0.030)	-1.23 (0.016)	-1.24 (0.054)
*pub count* = 1	-0.24 (0.005)	-0.16 (0.017)	-0.19 (0.011)	-0.19 (0.035)
*log*_10_(*pub count* + 1)	1.61 (0.007)	1.91 (0.020)	2.17 (0.008)	2.47 (0.022)
*log*_10_(*pub count* + 1)^2^	-0.09 (0.002)	-0.16 (0.006)	-0.34 (0.002)	-0.40 (0.006)
**pub type**				
Journal	0.27 (0.011)	-	0.23 (0.010)	-
Review	-0.08 (0.003)	-	-0.25 (0.002)	-
Case Report	-0.94 (0.007)	-	-0.95 (0.005)	-
Letter	-0.43 (0.011)	-	-0.34 (0.010)	-
ref = Journal	0.33 (0.011)	0.50 (0.032)	0.32 (0.009)	0.58 (0.027)
ref = Review	-0.78 (0.003)	-0.64 (0.007)	-0.64 (0.002)	-0.64 (0.006)
ref = Case Report	-0.64 (0.006)	-0.86 (0.017)	-0.73 (0.005)	-1.05 (0.014)
ref = Letter	-0.28 (0.011)	-0.45 (0.031)	-0.29 (0.009)	-0.37 (0.026)
**time lag**				
*timelag* < 0	-0.56 (0.016)	-0.33 (0.041)	-0.38 (0.015)	-0.39 (0.044)
*timelag* = 0	0.54 (0.006)	0.58 (0.015)	0.74 (0.005)	0.75 (0.013)
*log*_10_(*timelag* + 1)	0.54 (0.015)	0.15 (0.038)	1.23 (0.011)	0.86 (0.029)
*log*_10_(*timelag* + 1)^2^	-1.28 (0.010)	-0.86 (0.023)	-1.40 (0.007)	-1.16 (0.017)
**venue**				
same journal	0.46 (0.003)	0.50 (0.007)	0.53 (0.002)	0.62 (0.005)
*journal similarity* = 0	-0.28 (0.008)	-0.16 (0.022)	-0.30 (0.006)	-0.19 (0.017)
*log*_10_(*journal similarity* + 1)	0.06 (0.010)	0.26 (0.027)	0.13 (0.008)	0.36 (0.022)
*log*_10_(*journal similarity* + 1)^2^	0.01 (0.003)*	-0.06 (0.008)	-0.05 (0.002)	-0.14 (0.007)
**references**				
*count* = 1	-0.30 (0.022)	-	-0.41 (0.021)	-
*log*_10_(*count*)	-1.65 (0.034)	-7.40 (0.816)	-2.22 (0.031)	-17.48 (1.420)
*log*_10_(*count*)^2^	0.82 (0.023)	5.40 (0.550)	1.46 (0.021)	13.42 (1.039)
*log*_10_(*count*)^3^	-0.23 (0.005)	-1.39 (0.122)	-0.39 (0.005)	-3.42 (0.251)
**country (vs. USA)**				
Unknown	-0.04 (0.006)	-	-0.16 (0.005)	-
UK	-0.01 (0.003) X	-	-0.09 (0.002)	-
Japan	0.27 (0.006)	-	-0.09 (0.006)	-
Germany	0.02 (0.004)	-	-0.17 (0.003)	-
France	-0.04 (0.005)	-	-0.12 (0.004)	-
Italy	-0.10 (0.007)	-	-0.30 (0.005)	-
Canada	0.00 (0.004) X	-	-0.07 (0.003)	-
China	-0.41 (0.010)	-	-0.43 (0.007)	-
Australia	0.09 (0.005)	-	-0.10 (0.004)	-
Spain	-0.12 (0.006)	-	-0.18 (0.005)	-
Netherlands	0.07 (0.007)	-	-0.18 (0.005)	-
Sweden	0.11 (0.007)	-	-0.08 (0.005)	-
India	-0.04 (0.010)	-	-0.21 (0.008)	-
Other	-0.06 (0.003)	-	-0.21 (0.002)	-
**novelty**				
*log*_10_(*score* + 1)	-0.11 (0.001)	-0.11 (0.004)	-0.14 (0.001)	-0.16 (0.003)
*log*_10_(*ref. score* + 1)	0.09 (0.008)	0.16 (0.021)	0.05 (0.005)	0.20 (0.014)
*log*_10_(*ref. score* + 1)^2^	-0.03 (0.001)	-0.04 (0.004)	-0.02 (0.001)	-0.05 (0.002)
**language**				
English	0.65 (0.007)	-	0.70 (0.007)	-
ref. English	-0.18 (0.010)	3.63 (0.268)	-0.03 (0.009)	3.96 (0.268)
**author count**				
*author count* > 20	0.00 (0.015) X	-0.05 (0.034) X	-0.05 (0.013)	-0.04 (0.032) X
*log*_10_(*author count*)	0.08 (0.018)	0.30 (0.045)	-0.44 (0.014)	-0.18 (0.035)
*log*_10_(*author count*)^2^	-0.26 (0.013)	-0.35 (0.033)	0.07 (0.010)	-0.09 (0.026)
**ethnicity (vs. ENGLISH)**				
GERMAN	-0.01 (0.004)	-	0.06 (0.003)	-
HISPANIC	0.05 (0.004)	-	0.08 (0.003)	-
CHINESE	0.05 (0.004)	-	0.03 (0.004)	-
JAPANESE	-0.10 (0.006)	-	-0.05 (0.006)	-
SLAV	0.04 (0.005)	-	0.03 (0.004)	-
FRENCH	-0.07 (0.004)	-	-0.04 (0.003)	-
ITALIAN	-0.06 (0.006)	-	0.03 (0.004)	-
INDIAN	-0.06 (0.005)	-	0.02 (0.004)	-
NORDIC	0.14 (0.005)	-	0.07 (0.003)	-
ARAB	-0.05 (0.006)	-	-0.09 (0.006)	-
DUTCH	0.03 (0.007)	-	0.00 (0.005) X	-
KOREAN	-0.19 (0.007)	-	-0.08 (0.006)	-
UNKNOWN	0.01 (0.018) X	-	-0.05 (0.014).	-
OTHER	-0.18 (0.004)	-	-0.06 (0.003)	-
**MeSH**				
*count* = 0	-0.67 (0.013)	-0.63 (0.036)	-0.89 (0.010)	-0.91 (0.028)
*ref. count* = 0	-0.09 (0.015)	-0.16 (0.044)	0.01 (0.011) X	-0.11 (0.030)
*log*_10_(*count* + 1)	-0.17 (0.005)	-0.15 (0.014)	-0.25 (0.004)	-0.24 (0.011)
*log*_10_(*ref. count* + 1)	-0.17 (0.006)	-0.26 (0.015)	-0.09 (0.004)	-0.23 (0.011)
**gender (vs. Unknown)**				
Female	-0.03 (0.003)	-0.03 (0.012).	0.03 (0.003)	0.03 (0.011).
Male	-0.04 (0.003)	-0.01 (0.012) X	0.00 (0.003) X	0.01 (0.010) X

^†^Format: logit (SE) signif., where X = *p* ≥ 0.05, * = *p* < 0.05, . = *p* < 0.01, and *p* < 0.001 otherwise.

## Discussion

The takeaways from the present study are threefold. First, models that lack sufficient controls jeopardize the conclusions drawn from them, potentially with adverse effects on public perception and public policy. Human social behavior is complex and, thus, unlikely to be explained adequately without a diverse set of controls. The concern becomes clear when we consider the dramatic change in gender’s effect in this study with the introduction of confounding factors. MacRoberts et al. [53] asserted the following: *“Today, in spite of an overwhelming body of evidence to the contrary, citation analysts continue to accept the traditional view of science as a privileged enterprise free of cultural bias and self-interest and accordingly continue to treat citations as if they were culture free measures.”*. If cultural bias and self-interest influence self-citations, they are perhaps more charitably regarded as aspects of opportunity, accessibility, and visibility. First and last author positions on a byline enhance the visibility of the authors who occupy them, even if the position itself indicates nothing about the relative contribution of the author [54]. However, if we assume that authors in the most prominent positions on a byline have more influence over citations in the manuscript, we should not have difficulty imagining that particular positions offer increased opportunity for self-citation. From the results herein, the opportunities likely depend on a reference’s accessibility (physical, economic) as well as the degree to which the citation enhances the cited’s visibility (in terms of the work’s topical relevance, despite specialization, or the author’s prominence).

**Fig 5:**
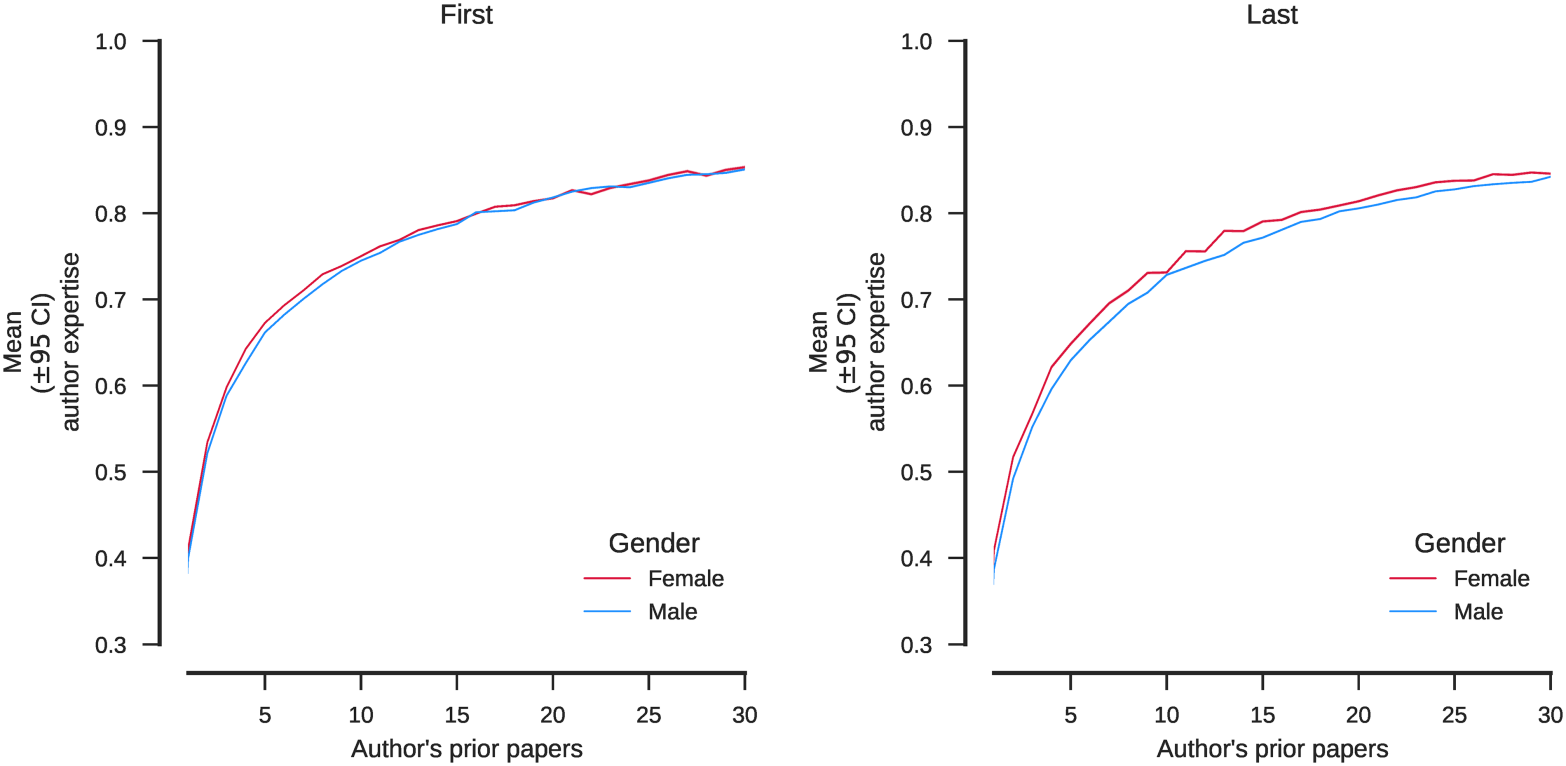
Author expertise as a function of prior publication count. Expertise of an author on a given paper is measured by the proportion of subjects (MeSH; a paper typically has a dozen or so terms) on which the author has previously published. Expertise naturally grows with age but never reaches 100% because authors tend to publish on some topics that are new to them.

Second, self-citation is the hallmark of highly productive authors, of any gender, who cite their novel journal publications early in similar venues. As a result, papers by authors with short, disrupted, or diverse careers lack the initial boost in visibility gained from self-citations. This disproportionately affect women who tend to leave (and enter) science at higher rates than men (see Table 8), and have different career trajectories for a variety of reasons [55]. Prior work has found that men specialize more than women and therefore can publish more [56]. However, in our dataset we find that men and women have the same age-normalized expertise, on average (Fig 5). In other words, attrition is the most likely driver of overall differences in self-citation rates, not topical specialization or citation strategies. Reducing attrition and achieving a more gender-balanced scientific workforce [57] is essential to improving scientific progress. It should help increase self-citation, a signal that more research is followed-up by the scientists best situated to do so.

**Table 8:** Percentage of authorships, on the 1.6 million articles with 2 or more authors published between 2002-2005, by authors who (a) started, (b) ended, and (c) started as well as ended their career in during period. Note that career start and end years were determined based on the full 2009 Author-ity dataset.

Ethnicity	First Author									Last Author								
	Started			Ended			Started—Ended			Started			Ended			Started—Ended		
	U	F	M	U	F	M	U	F	M	U	F	M	U	F	M	U	F	M
ENGLISH	44.1	33.1	22.9	36.7	20.6	13.9	24.5	10.9	6.4	33.2	17.4	7.2	38.4	16.8	8.7	26.5	9.3	3.6
GERMAN	50.7	33.1	18.8	50.4	19.4	10.5	34.0	9.6	4.2	38.0	13.1	4.4	48.1	13.1	5.9	31.8	6.3	1.7
HISPANIC	56.5	34.6	26.1	48.6	17.4	11.9	33.9	9.4	6.1	43.9	15.9	8.5	52.8	13.1	8.2	35.1	7.3	3.7
CHINESE	45.2	43.4	41.0	14.9	16.1	13.6	8.8	9.0	7.6	23.6	22.7	17.6	11.9	12.6	8.1	7.4	7.3	4.6
JAPANESE	25.3	31.8	19.7	18.7	23.1	13.8	9.1	10.3	5.1	9.2	15.5	5.0	13.8	16.3	7.7	6.2	7.6	2.1
SLAV	35.8	29.4	22.1	35.7	16.1	10.0	20.0	7.7	4.1	33.9	19.4	8.6	43.5	17.9	8.5	24.6	9.4	3.3
FRENCH	44.0	30.6	21.1	38.7	16.0	9.8	24.6	7.4	4.0	25.5	9.0	4.0	34.4	9.6	5.6	19.6	4.0	1.6
ITALIAN	45.6	19.3	11.8	46.2	9.9	6.0	29.6	4.1	2.2	37.9	11.0	4.3	49.5	9.4	5.5	31.4	4.3	1.6
INDIAN	41.8	41.2	35.1	23.9	19.0	13.3	15.1	10.6	7.0	20.9	20.7	12.5	20.6	14.9	8.4	11.8	8.6	3.8
NORDIC	42.9	31.8	21.1	36.0	18.6	11.4	23.2	8.0	4.4	26.3	8.8	3.9	33.1	9.8	5.6	22.0	4.3	1.7
ARAB	46.3	42.9	32.8	33.0	22.2	15.2	22.1	12.4	7.7	38.5	31.9	17.8	37.2	24.5	13.8	27.3	16.0	7.5
DUTCH	41.1	33.4	20.8	30.4	15.9	9.8	17.8	6.2	3.6	16.1	9.3	3.1	22.2	9.6	4.4	12.9	4.4	1.4
KOREAN	41.5	38.3	35.2	15.6	13.0	9.6	10.1	7.8	5.2	17.0	19.2	14.5	10.2	9.3	6.2	6.2	6.4	3.2
UNKNOWN	55.5	41.5	28.0	40.6	24.5	16.7	29.9	14.8	7.2	36.3	19.7	10.1	46.1	18.6	12.1	29.4	11.6	5.3
OTHER	38.8	32.3	22.2	25.2	14.7	8.5	16.1	7.8	3.8	28.1	19.5	9.4	28.0	13.6	7.7	17.8	7.6	3.0
OVERALL	43.0	32.6	22.9	23.6	18.0	11.9	14.8	9.1	5.2	24.9	15.8	6.8	23.0	14.3	7.5	14.5	7.6	2.8

Third, the scientometric practice of discounting self-citations has the adverse effects of penalizing so-called lines of research and reducing the impact of some important papers already suffering from lower discoverability and accessibility because of non-English language, lack of bibliographic indexing, and so on. Authors most likely to be affected by such a penalty are perhaps the most dependent on sponsorship and patronage in science [58, 59]. Citations reflect potentially many different authorial attitudes: to credit the source of inspiration; to aid the understanding of the reader; to assert authority in a field [48]; but self-citations acknowledge an individual’s line of research. One might worry about “salami-slicing” [60] and disciplinary “siloing” [61] but one should also hope that scholars conduct research in the way that is effective for them individually. Tempering labels like being “your own favorite expert” [17] would be a good start. After all, a persistent lack of (self-)citations likely reflects dead-ends and orphans not even nurtured by the scholars with a vested interest.

## Supporting information

S1 Fig Self-citation rates as functions of author age as measured by prior publication count (top panels) for the second-listed authors on papers with three or more authors. The horizontal lines show the overall self-citation rates. The bottom panels show the cumulate distributions of author age.
(PDF)

S2 Fig Self-citation rates as functions of author age for journals from science category. The horizontal lines show the overall self-citation rates.
(PDF)

S3 Fig Self-citation rates as functions of author age for journals from biology category. The horizontal lines show the overall self-citation rates.
(PDF)

S4 Fig Self-citation rates as functions of author age for journals from medicine category. The horizontal lines show the overall self-citation rates.
(PDF)

S5 Fig Plots of empirical vs. fitted values for select predictors from the complete models for (a) first and (b) last author data. For each plot, empirical data are represented by bubbles, the size of which are proportional to the number of data points each contains; bubble color reflects the number of actual self-citations denoted in the accompanying legend. Red lines show the fit for a predictor given all terms in the complete model. The alignment of bubbles and lines provides evidence that the chosen modeling framework (logistic regression, a linear model) is appropriate.
(PDF)

S6 Fig Receiver Operating Curve (ROC) and Precision Recall Curves (PRC) for complete first and last author models. A model that fit the data perfectly would hug the upper left and upper right corners of the ROC and PRC plots, respectively. A model no better than random would hug the thin gray diagonal line.
(TIF)
