# Supplementary figures and images for: Self-citation is the hallmark of productive authors, of any gender
Source: PLoS One. 2018 Sep 26;13(9):e0195773. doi: 10.1371/journal.pone.0195773 (PMC6157831; doi:10.1371/journal.pone.0195773)

## Middle 2nd

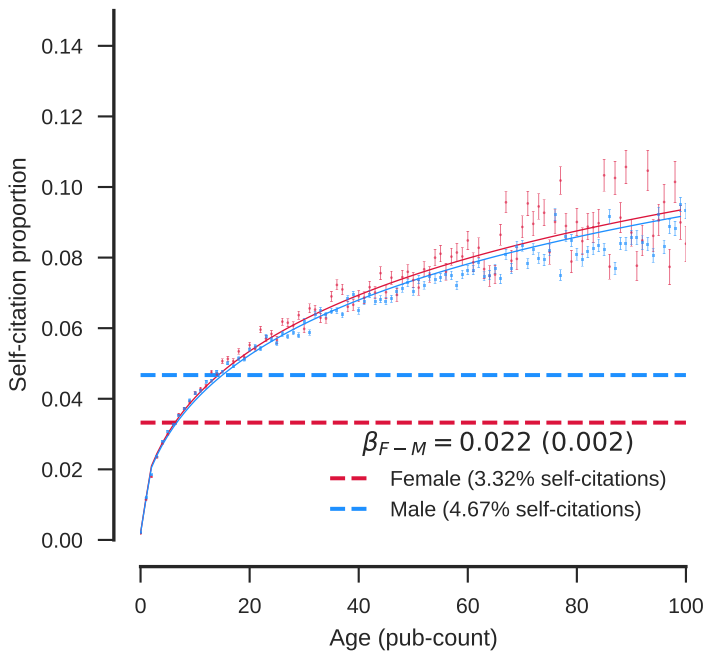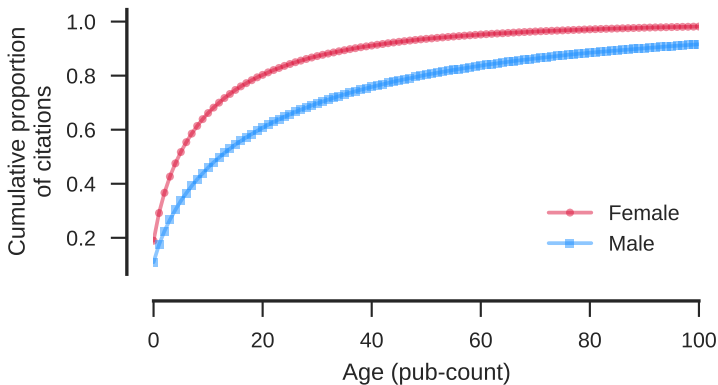

Supplement: S1 Fig — The horizontal lines show the overall self-citation rates. The bottom panels show the cumulate distributions of author age. (PDF) [file pone.0195773.s001.pdf]

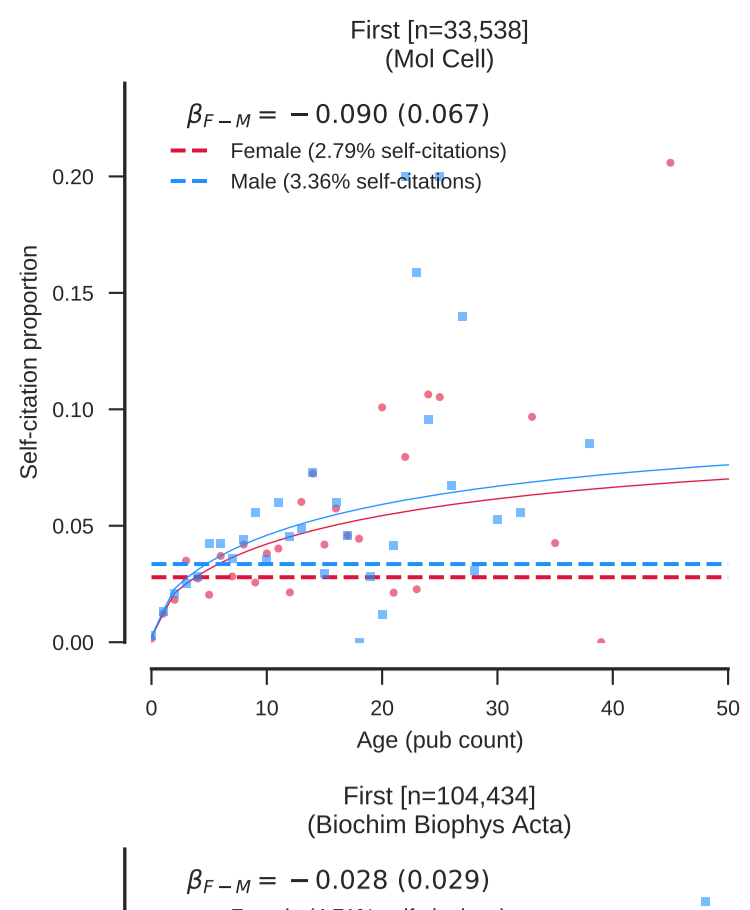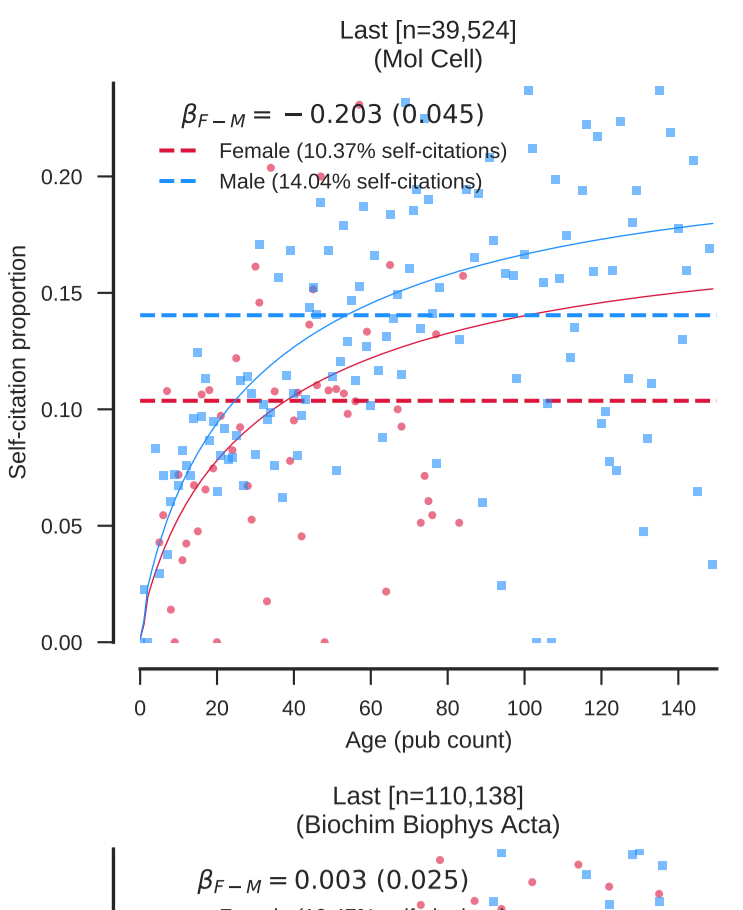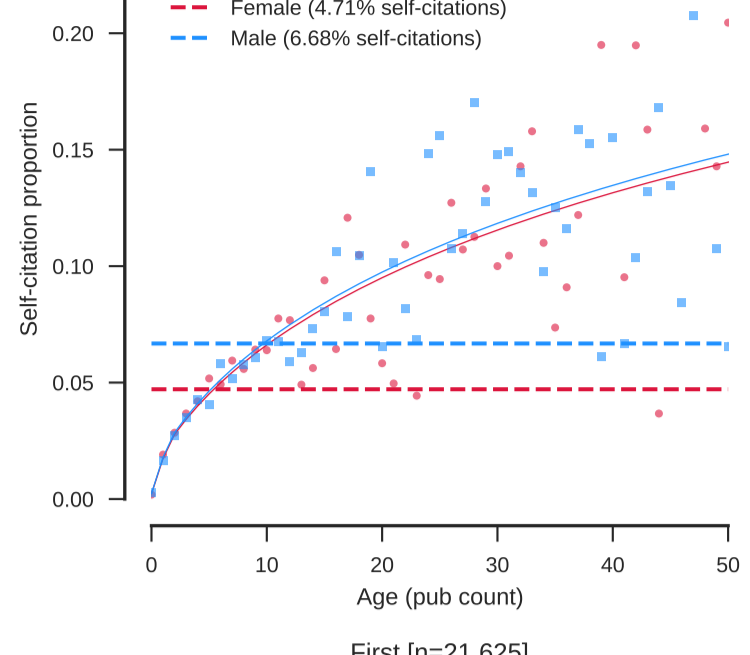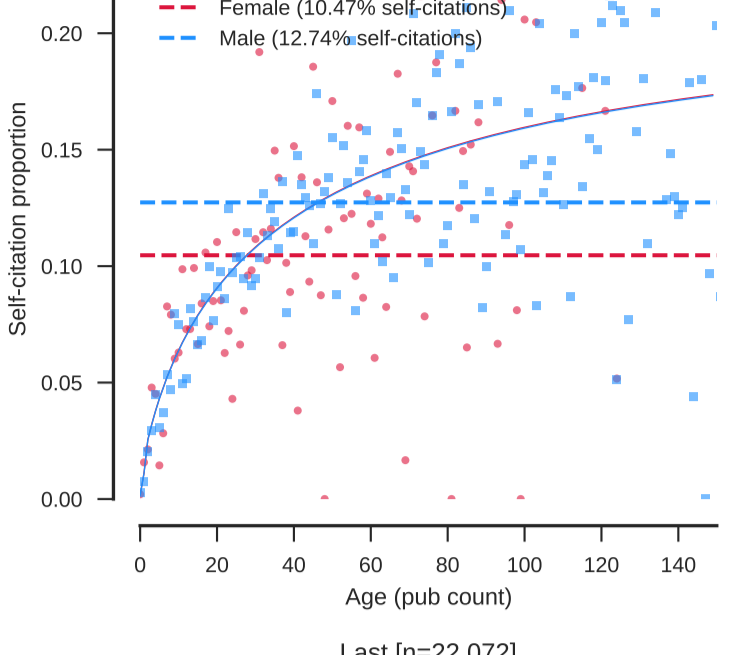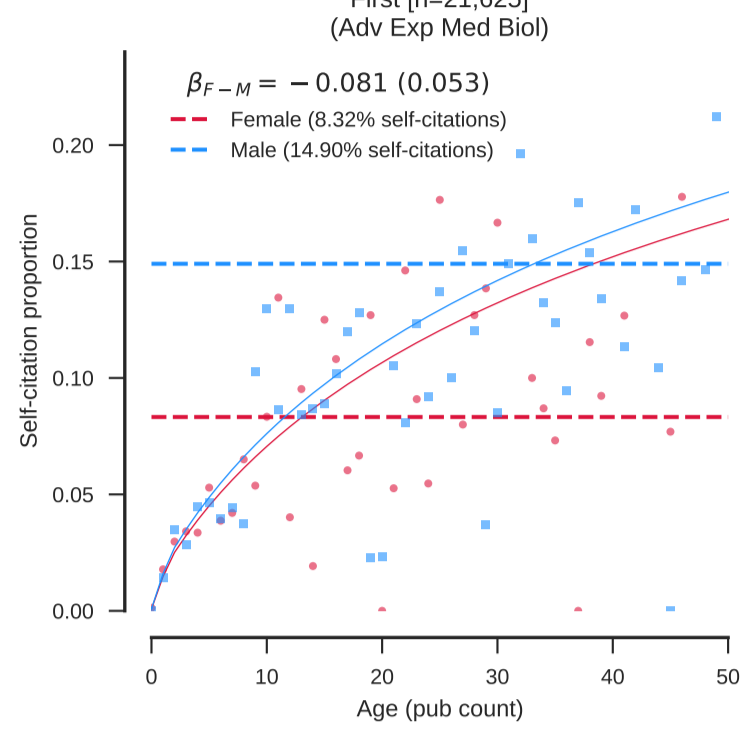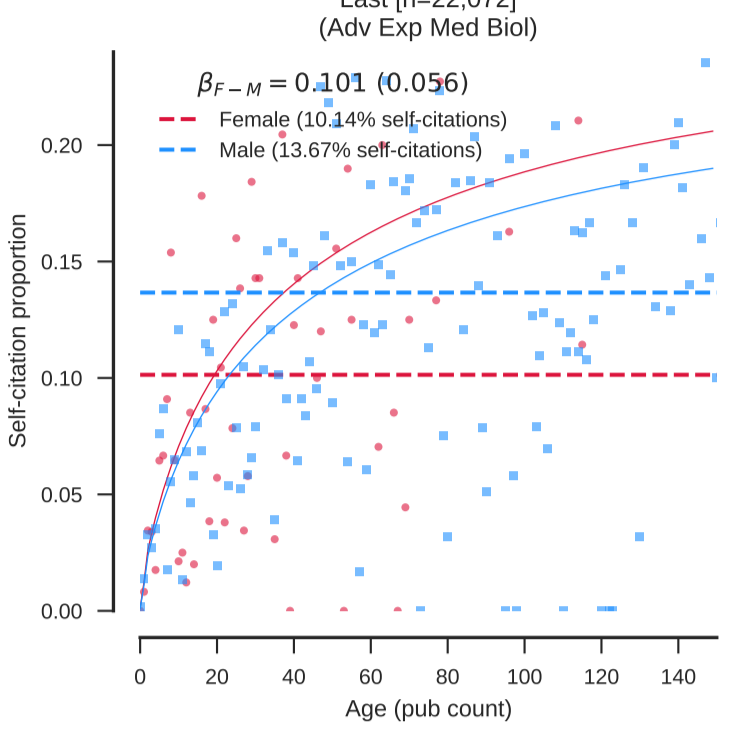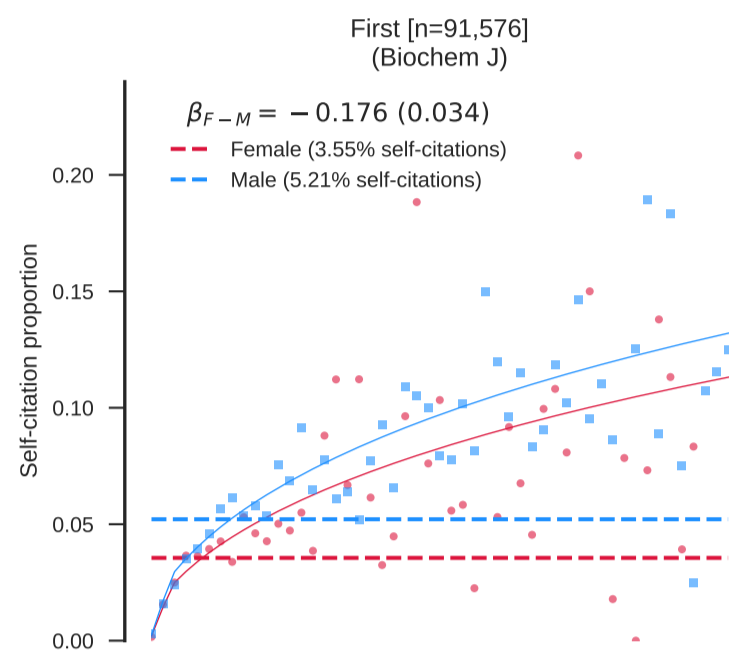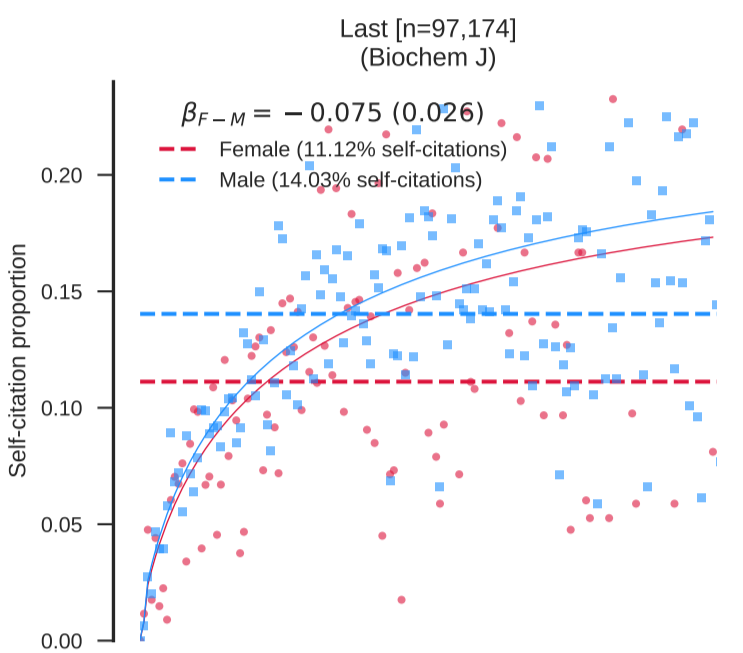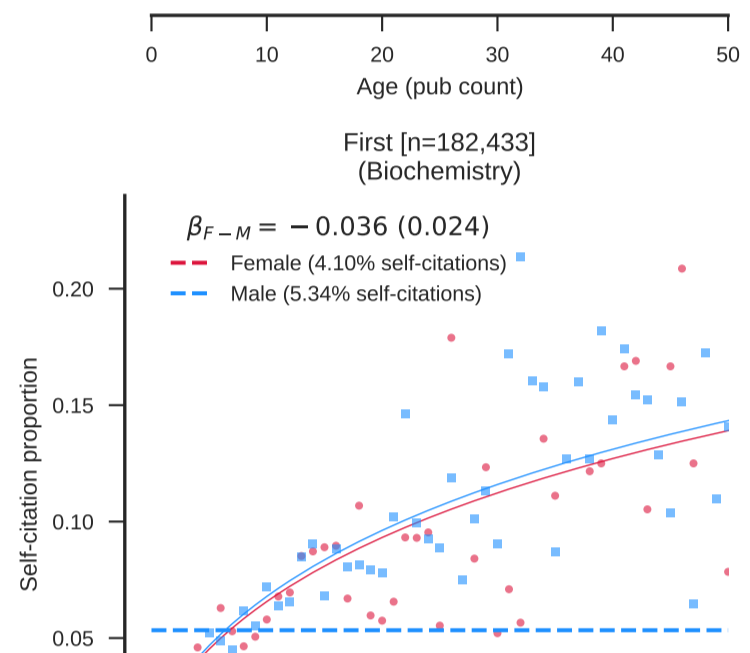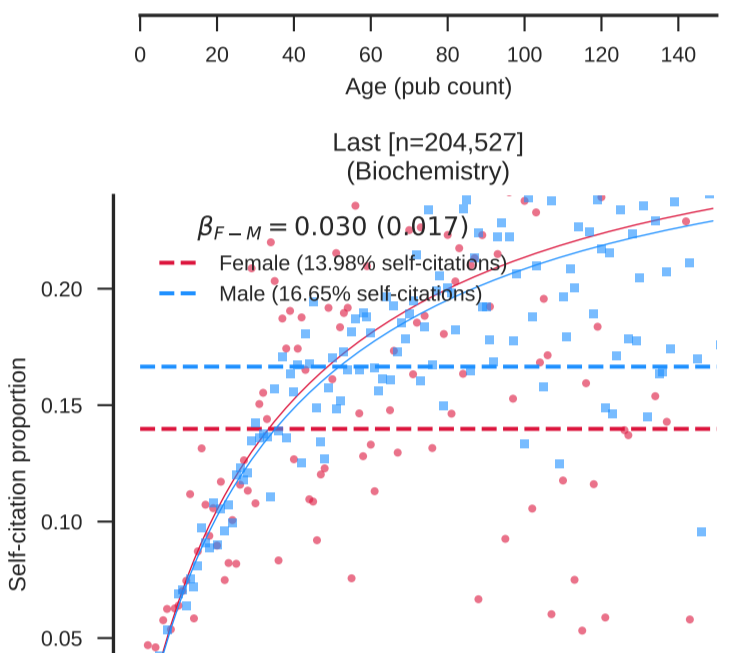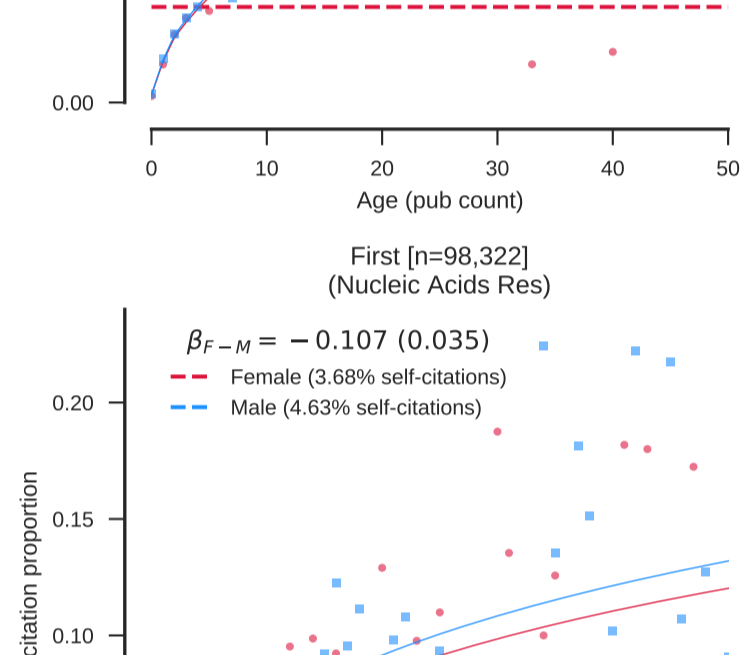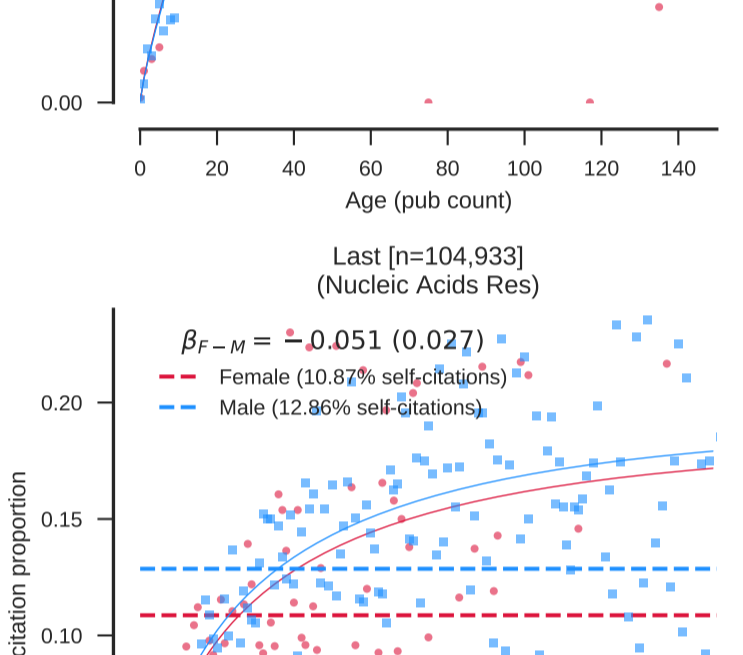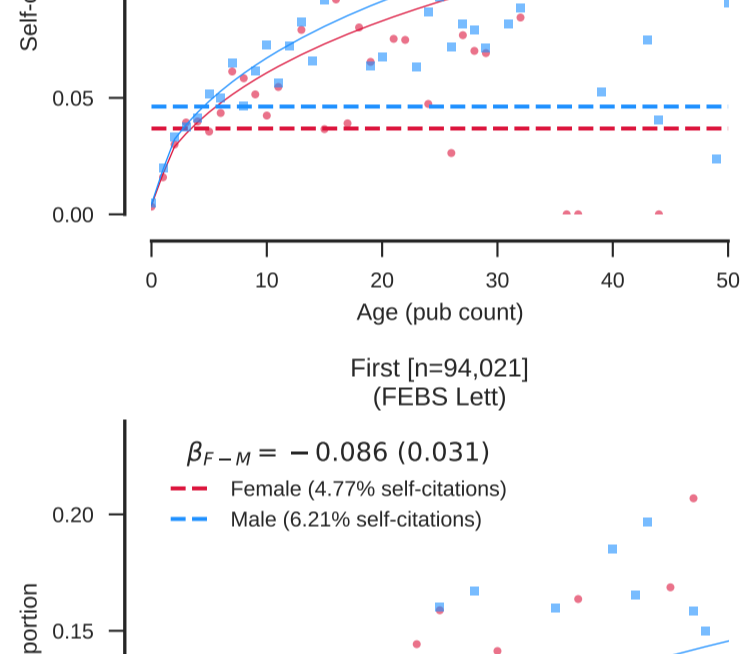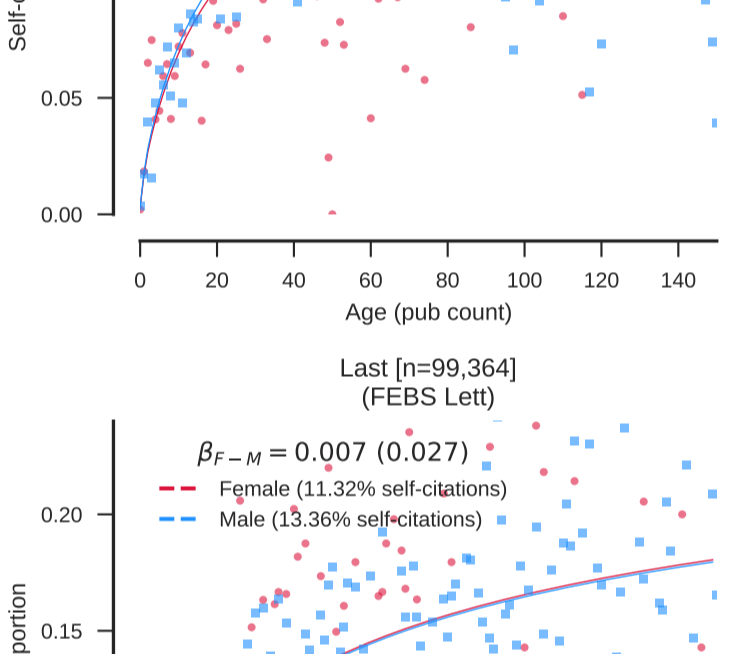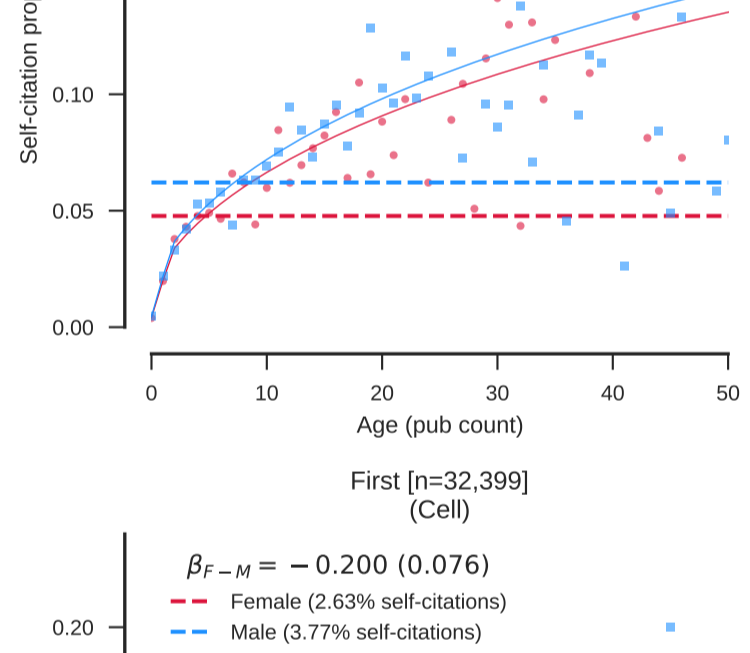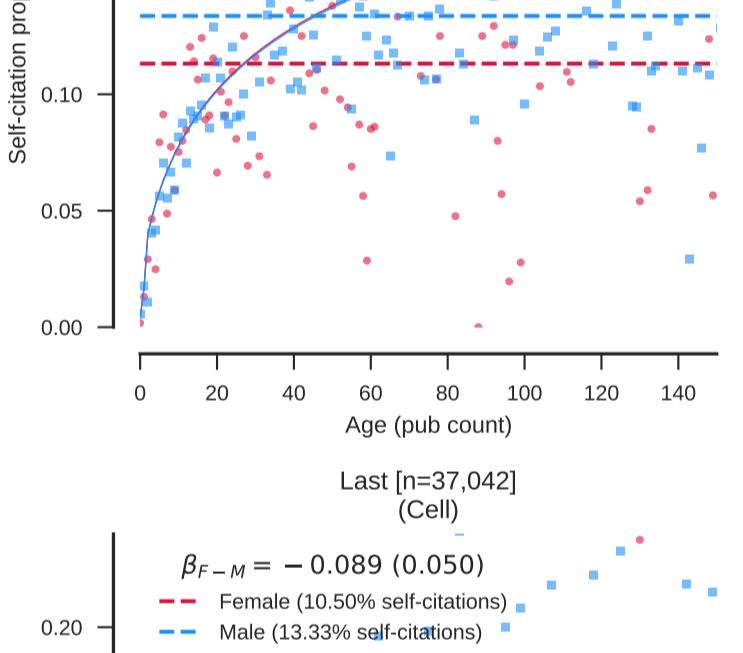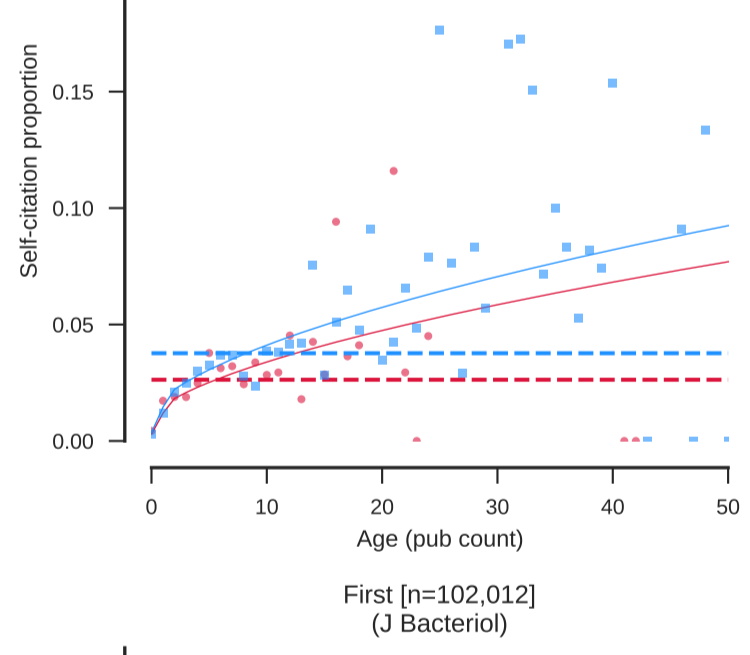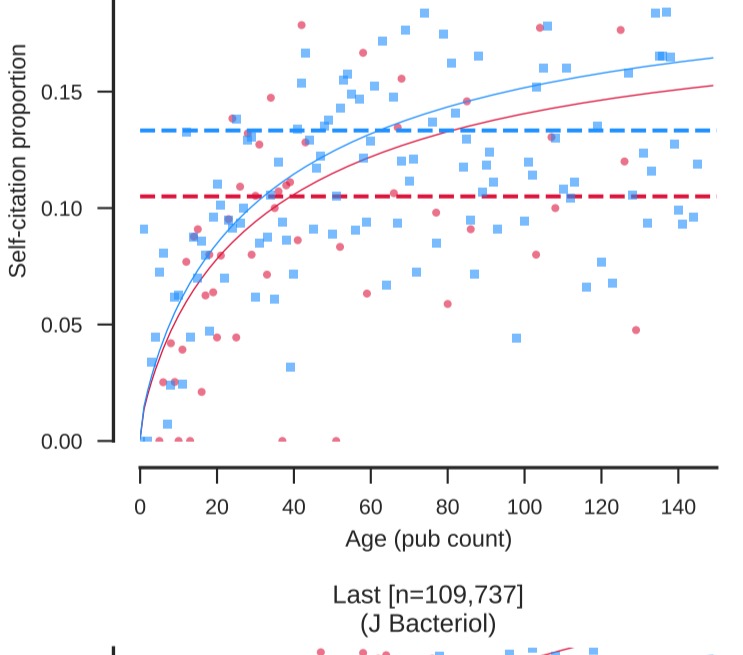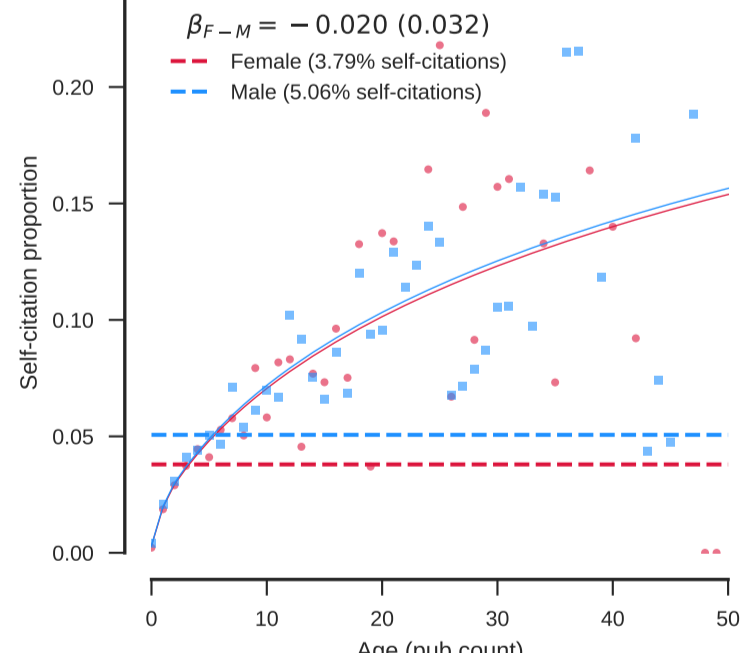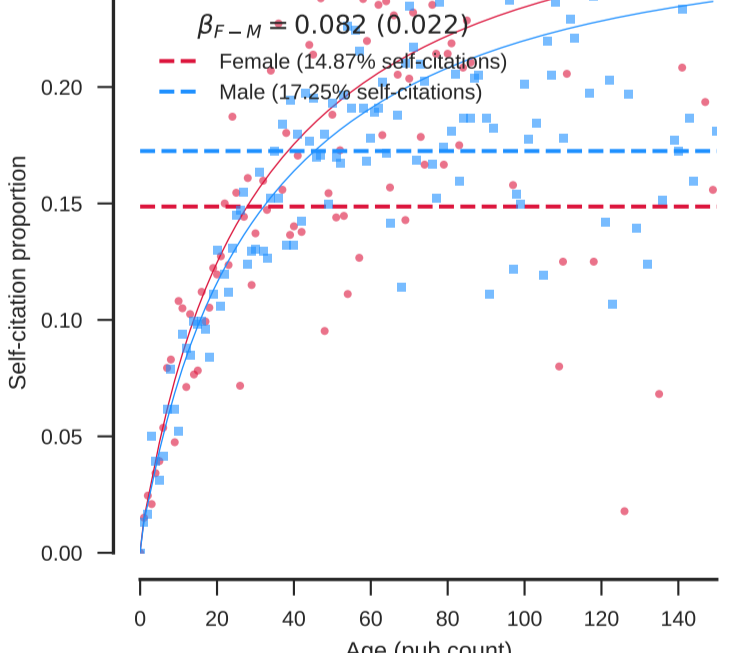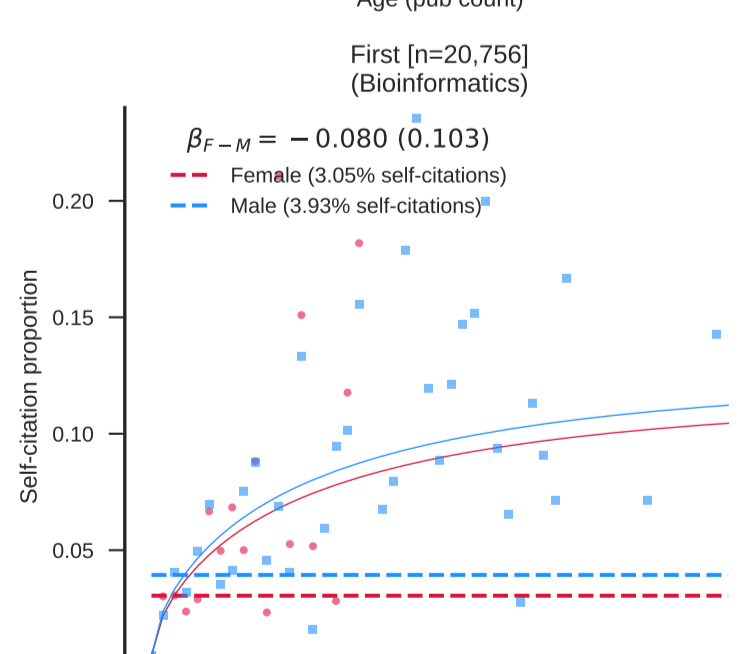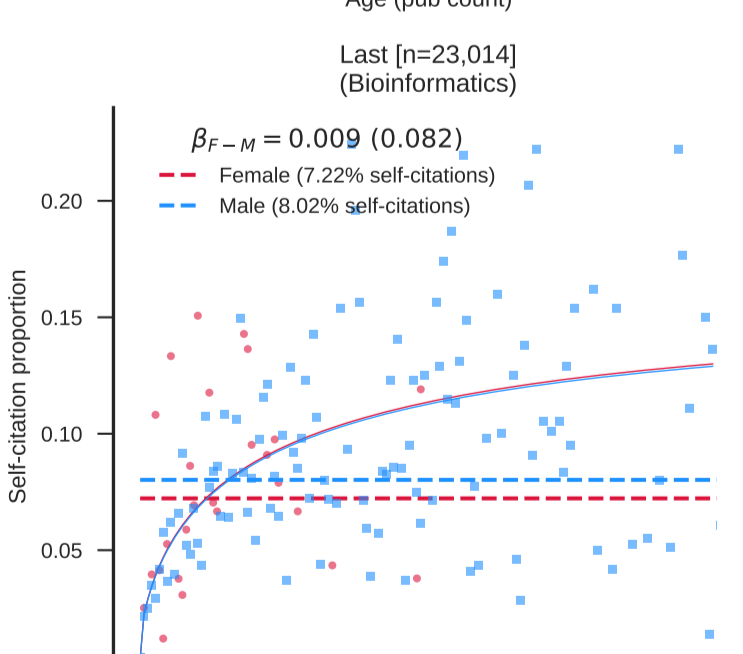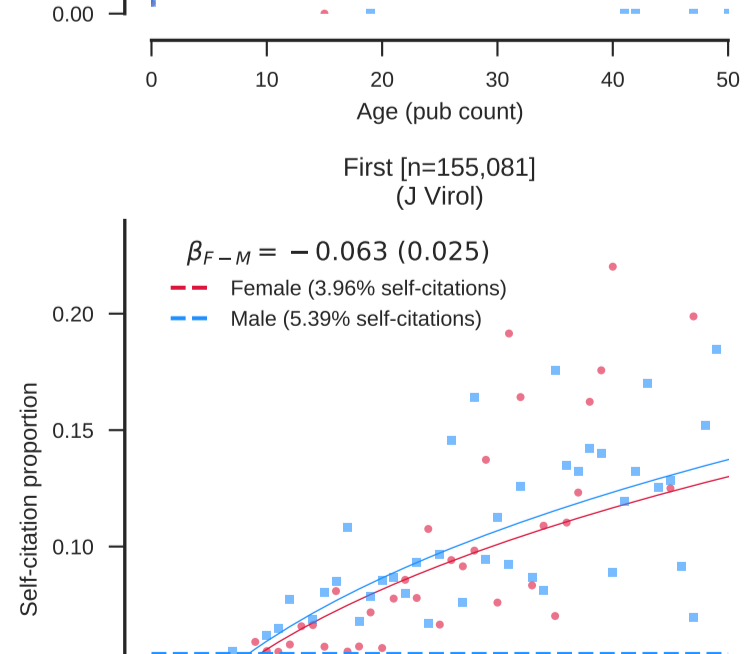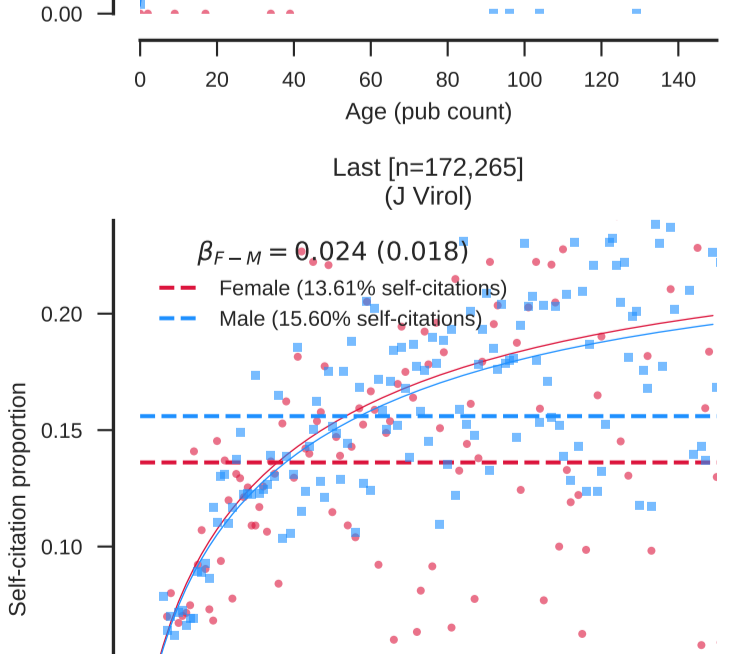

Supplement: S3 Fig — The horizontal lines show the overall self-citation rates. (PDF) [file pone.0195773.s003.pdf]

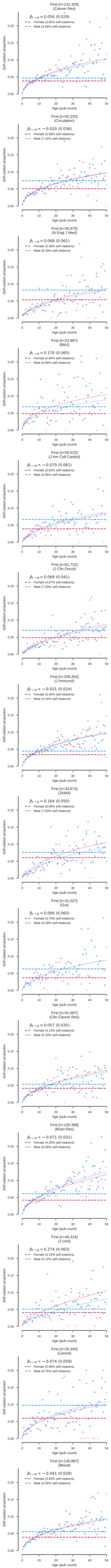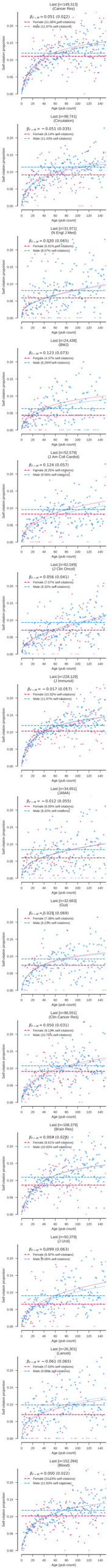

Supplement: S4 Fig — The horizontal lines show the overall self-citation rates. (PDF) [file pone.0195773.s004.pdf]

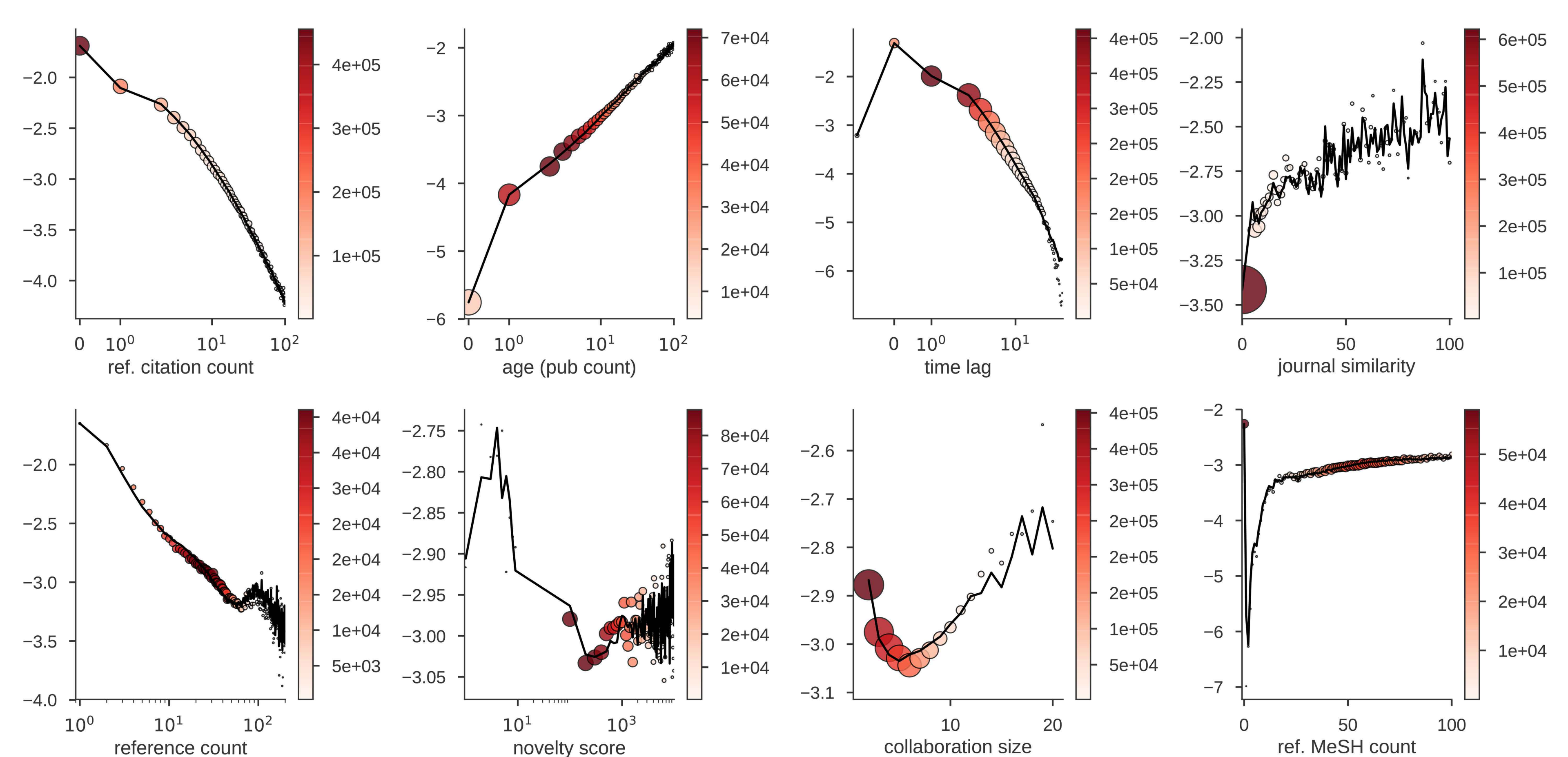

(a) First author

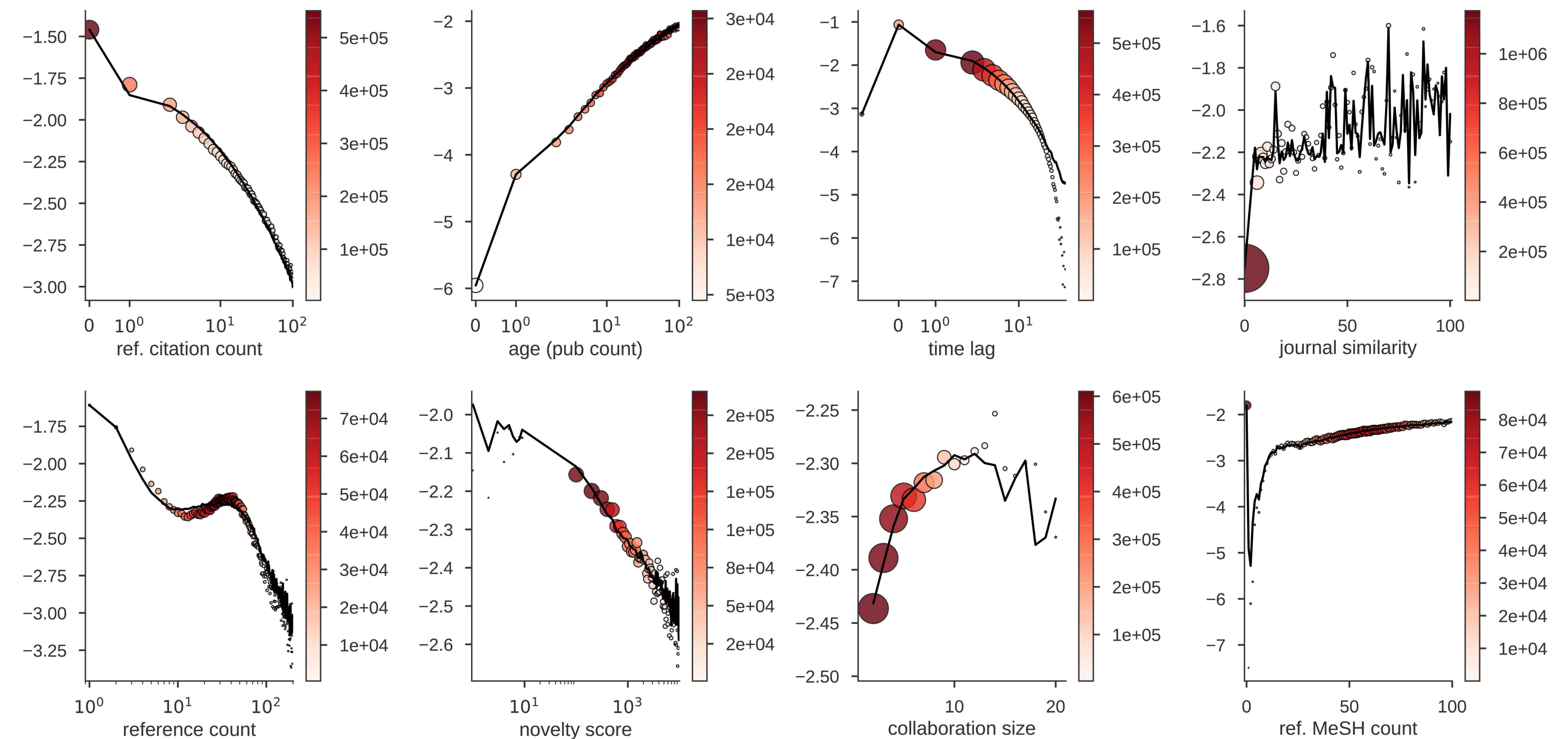

(b) Last author

Supplement: S5 Fig — For each plot, empirical data are represented by bubbles, the size of which are proportional to the number of data points each contains; bubble color reflects the number of actual self-citations denoted in the accompanying legend. Red lines show the fit for a predictor given all terms in the complete model. The alignment of bubbles and lines provides evidence that the chosen modeling framework (logistic regression, a linear model) is appropriate. (PDF) [file pone.0195773.s005.pdf]

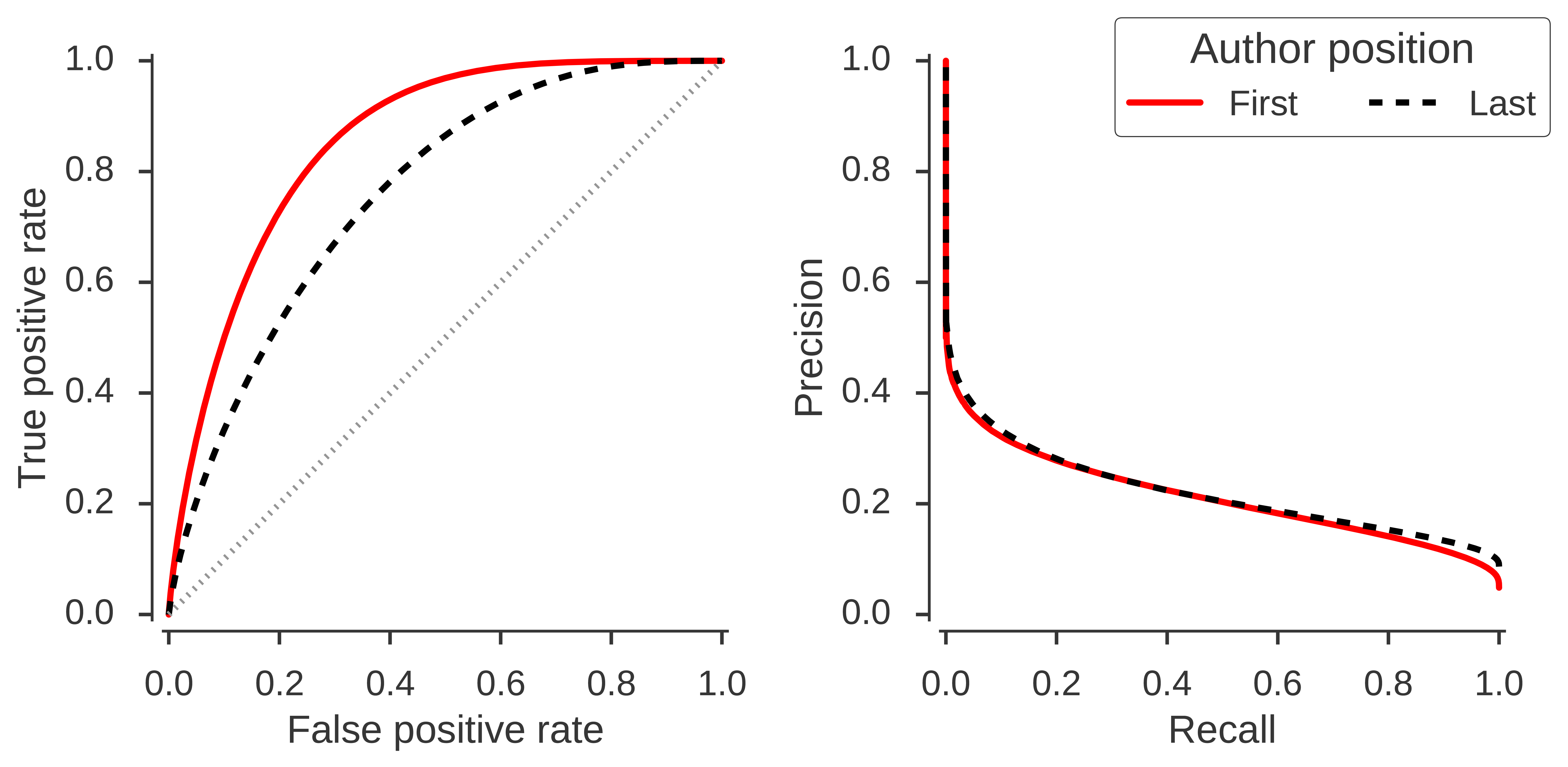

Supplement: S6 Fig — A model that fit the data perfectly would hug the upper left and upper right corners of the ROC and PRC plots, respectively. A model no better than random would hug the thin gray diagonal line. (TIF) [file pone.0195773.s006.tif]
