# Supplementary material for: Self-citation is the hallmark of productive authors, of any gender
Source: PLoS One. 2018 Sep 26;13(9):e0195773. doi: 10.1371/journal.pone.0195773 (PMC6157831; doi:10.1371/journal.pone.0195773)
Supplement: S2 Fig — The horizontal lines show the overall self-citation rates. (PDF) [file pone.0195773.s002.pdf]

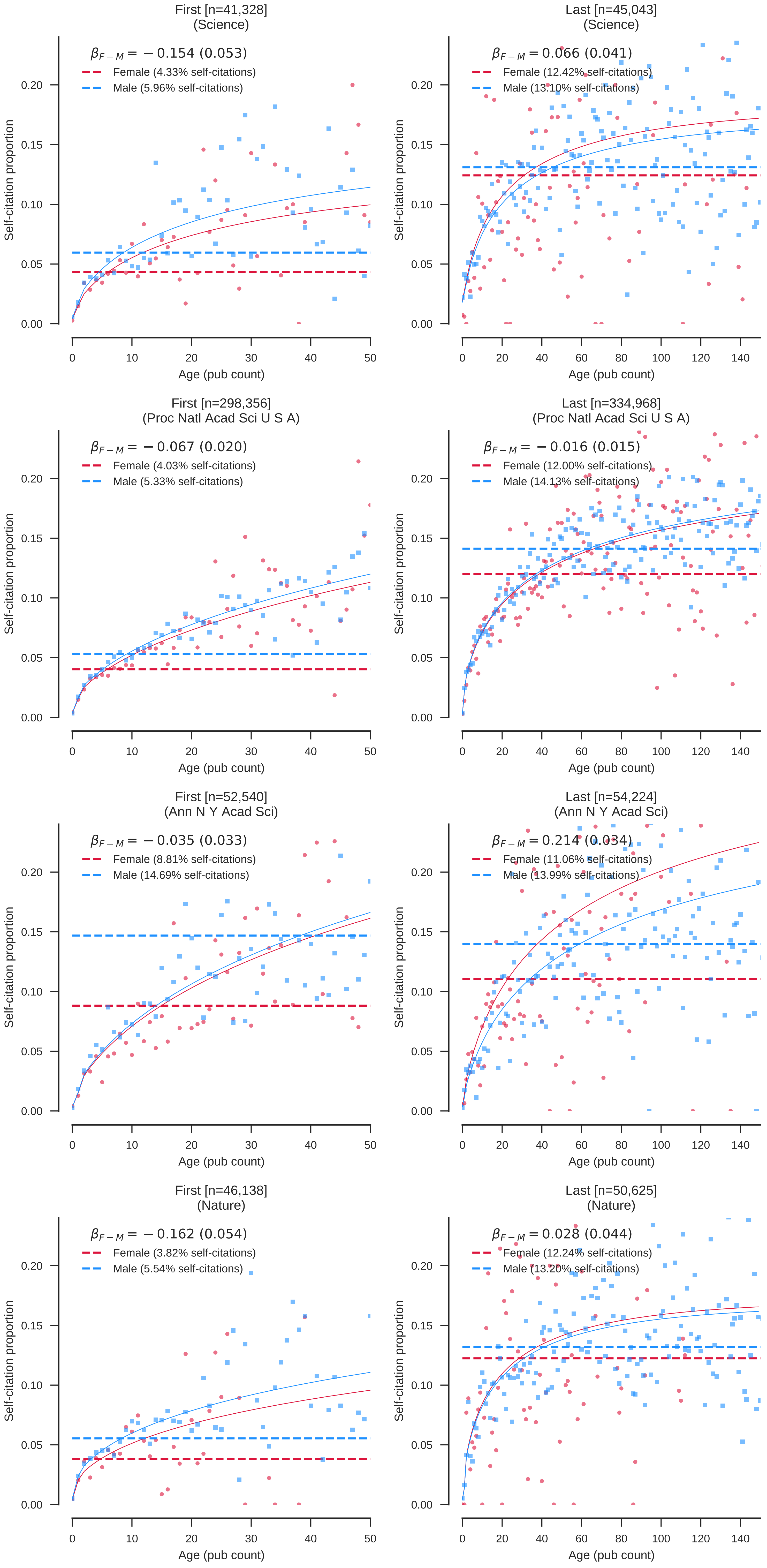

First [n=41,328]
(Science)
$\beta_{F-M} = -0.154$ (0.053)
Female (4.33% self-citations)
Male (5.96% self-citations)
Last [n=45,043]
(Science)
$\beta_{F-M} = 0.066$ (0.041)
Female (12.42% self-citations)
Male (13.10% self-citations)
First [n=298,356]
(Proc Natl Acad Sci U S A)
$\beta_{F-M} = -0.067$ (0.020)
Female (4.03% self-citations)
Male (5.33% self-citations)
Last [n=334,968]
(Proc Natl Acad Sci U S A)
$\beta_{F-M} = -0.016$ (0.015)
Female (12.00% self-citations)
Male (14.13% self-citations)
First [n=52,540]
(Ann N Y Acad Sci)
$\beta_{F-M} = -0.035$ (0.033)
Female (8.81% self-citations)
Male (14.69% self-citations)
Last [n=54,224]
(Ann N Y Acad Sci)
$\beta_{F-M} = 0.214$ (0.034)
Female (11.06% self-citations)
Male (13.99% self-citations)
First [n=46,138]
(Nature)
$\beta_{F-M} = -0.162$ (0.054)
Female (3.82% self-citations)
Male (5.54% self-citations)
Last [n=50,625]
(Nature)
$\beta_{F-M} = 0.028$ (0.044)
Female (12.24% self-citations)
Male (13.20% self-citations)
Self-citation proportion
Age (pub count)
